# Development and Temporal External Validation of a Parsimonious, Interpretable Machine Learning Model for Predicting 6-Month Mortality in Long-Term Care Facilities: A Retrospective Cohort Study

**DOI:** 10.2196/94567

**Published:** 2026-09-16

**Authors:** Yun-Cheng Tsai, Yi-Lin Wu, Yung-Chen Yu, Yi-Wen Chen, Yi-Wen Chen, Yi-Ching Yang, Yu-Tai Lo

**Affiliations:** 1 PecuLab LLC, Seattle, WA, United States; 2 Department of Nursing, National Cheng Kung University Hospital, College of Medicine, National Cheng Kung University, Tainan, Taiwan; 3 Department of Nursing, College of Medicine, National Sun Yat-sen University, Kaohsiung, Taiwan; 4 Department of Business Development, Smart Ageing Tech Co., Ltd., New Taipei City, Taiwan; 5 Department of Family Medicine, College of Medicine, National Cheng Kung University, Tainan, Taiwan; 6 Department of Geriatrics and Gerontology, National Cheng Kung University Hospital, College of Medicine, National Cheng Kung University, No.138, Sheng Li Road, Tainan, 704, Taiwan, 886 6-2353535 ext 3828, 886 6-2083714; 7 Department of Public Health, College of Medicine, National Cheng Kung University, Tainan, Taiwan

**Keywords:** long-term care facilities, mortality prediction, machine learning, AI, Taiwan

## Abstract

**Background:**

Early mortality after long-term care facility (LTCF) admission is common; yet, prognostic tools are often derived from Western minimum dataset-based cohorts or require hospital electronic health record linkages that are unavailable at intake in many LTCFs. There is also limited evidence on explainable, admission-feasible machine learning–based prognostication in Asian LTCF settings.

**Objective:**

The aim of the study is to develop and temporally externally validate an interpretable machine learning model for predicting 6-month all-cause mortality among older adults newly admitted to LTCFs in Taiwan using routinely collected LTCF assessment data.

**Methods:**

We conducted a retrospective cohort study using the JUBO Long-Term Care Database, a nationwide private administrative registry covering 636 LTCFs (37.4% of the national LTCFs) in Taiwan. We included residents with first-time LTCF admission and prespecified nonoverlapping cohorts for temporal validation: development (January 1, 2020, to December 31, 2023; n=23,901) and external validation (January 1 to December 31, 2024; n=6216). The outcome measure was death within 180 days of admission. We compared a nonlinear ensemble model (hybrid of extreme gradient boosting and random forest [HybridXGBRF]) with 7 other algorithms, including tree-based and linear benchmarks. Discrimination (area under the receiver operating characteristic curve [AUROC]), classification metrics (accuracy, precision, recall, and *F*_1_), and calibration (Brier score and calibration plots) were assessed. Model interpretability was examined using Shapley Additive Explanations.

**Results:**

In the development cohort, 5272 of 23,901 (22.1%) residents died within 180 days. In the 2024 temporal validation cohort, 1781 of 6216 (28.7%) residents died. In internal cross-validation, HybridXGBRF had the highest AUROC among the evaluated models (0.89, 95% CI 0.88‐0.89). In temporal validation, HybridXGBRF maintained strong discrimination (AUROC 0.90, 95% CI 0.89‐0.91), with an accuracy of 0.85 and an *F*_1_-score of 0.68. Calibration plots indicated close agreement between predicted and observed risks across most probability ranges, with mild divergences at higher predicted risks. Shapley Additive Explanations analysis identified frequent hospitalizations within 6 months, activities of daily living impairment, and weight loss as influential predictors. The model showed stable AUROC across sex and age strata (0.89‐0.90) and maintained high discrimination among residents with improving activities of daily living scores (AUROC 0.91, 95% CI 0.90‐0.92).

**Conclusions:**

An interpretable machine learning model using routinely collected Taiwanese LTCF assessment data achieved strong discrimination, acceptable calibration, and stable temporal validation performance without requiring hospital-based electronic health record linkage. The HybridXGBRF had the highest AUROC among the evaluated models, but performance differences from extreme gradient boosting were small. The model may serve as a risk-stratification tool to help identify residents who could benefit from structured care review, advance care planning, or palliative care assessment. Prospective implementation studies could determine its impact on care processes and resident- or family-centered outcomes.

## Introduction

The accelerating population aging at a global scale has established long-term care facilities (LTCFs) as cornerstones of geriatric health care systems [1,2]. For many older adults, LTCF transition is a major event in their health trajectory, often marked by precipitous functional decline and limited life expectancy [1,3]. This renders paramount accurate prognostic assessment at admission, not only to align clinical care with patient goals but also to facilitate timely advance care planning (ACP), optimize resource allocation, and ensure equitable access to palliative services [4,5]. Substantial early mortality has been reported after first-time LTCF admission, with approximately 1 in 5 residents dying within the first months of placement, and mortality rates markedly exceeding those in age-matched community populations [2,3]. Therefore, there is importance in promoting the early identification of LTCF residents at high risk for short-term mortality.

Several prognostic tools have been developed to estimate mortality risk among LTCF residents, mostly in the United States and predominantly using a minimum dataset (MDS) or related assessment instruments [5-10]. Examples include the Advanced Dementia Prognostic Tool, designed for residents with advanced dementia [7,9], and the MDS Mortality Risk Index for 6-month risk stratification [5,6,8]. Although several of these models have undergone prospective validation [6,7], most were developed more than a decade ago and may not reflect contemporary care practices, evolving resident acuity, or shifts in LTCF populations following postacute care policy changes. Recent initiatives have attempted to address these limitations, achieving mixed success. The Hospice Eligibility Prediction Index incorporates various clinical indicators of hospital admission history [11], while Jorissen et al [12] developed a comprehensive 51-variable model with individual-, medication-, system-, and health care–related factors known at entry into an LTCF. However, their clinical utility remains hindered by excessive complexity and a lack of external validation in a greater number of populations.

In such contexts, machine learning methods offer potential advantages for risk prediction, including the ability to model nonlinear associations and higher-order interactions within correlated clinical domains [13]. Recent reports have described improved discrimination when using machine learning models that incorporate demographic characteristics, comorbidities, and prior health care use [14,15]. Despite the potential, many of the extant machine learning models for mortality prediction remain dependent on acute care hospitalization data, which are often inaccessible or significantly delayed during the LTCF intake process. Furthermore, evidence of AI-driven prognosis in Asian LTCF contexts remains sparse, leaving a critical gap in health equity and geographical generalizability [16].

To address these gaps, this study aimed to, first, develop an interpretable machine learning prediction model to predict 6-month mortality among older adults newly admitted to LTCFs in Taiwan, using variables that are routinely collected at the time of admission or routine early-stay assessments. Second, to temporally externally validate the model using a later calendar-year cohort.

## Methods

### Study Design, Dataset, and Population

Taiwan’s long-term care system provides a continuum of home-based, community-based, and institutional services for its rapidly aging population [17]. Residential LTCFs represent an institutional care setting within this continuum, serving older adults who require continuous assistance, supervision, nursing support, or help with activities of daily living (ADL) [18]. Accordingly, Taiwanese LTCF residents generally represent a frail and care-dependent population with substantial functional impairment, diverging from the broader community-dwelling older adult population. This study therefore focused on newly admitted LTCF residents, a clinically relevant point at which risk stratification may inform care planning, family communication, and ACP.

As aforementioned, we conducted a retrospective cohort study to develop and validate a machine learning model for predicting 6-month all-cause mortality among older adults newly admitted to LTCFs in Taiwan. This study used deidentified administrative and clinical assessment data routinely collected at the time of LTCF admission. Data were extracted from the JUBO Long-Term Care Database, a nationwide private administrative registry that captures structured admission, discharge, and clinical assessment data from LTCFs in Taiwan. The JUBO LTCFs system plays a significant role in Taiwan’s long-term care market, with its services being used by 37.4% of all LTCFs nationwide (ie, a total of 636 institutions). Most (67.7%) of the facilities were in the south, 20% were in the central region, 11% were in the north, and a small number were in the east. This study was reported in accordance with the TRIPOD-AI (Transparent Reporting of a Multivariable Prediction Model for Individual Prognosis Or Diagnosis-Artificial Intelligence) statement (Checklist 1) [19].

The study sample comprised all residents with a first-time LTCF admission recorded in the JUBO database between January 1, 2020, and December 31, 2024. First-time admission was defined as the first LTCF admission recorded for a resident in the JUBO database during the study period. To enable temporal external validation, we prespecified 2 nonoverlapping cohorts by admission date, as follows: a model development cohort including admissions from January 1, 2020, to December 31, 2023 (n=23,901); a temporal external validation cohort including admissions from January 1, 2024, to December 31, 2024 (n=6216).

**Figure 1.**
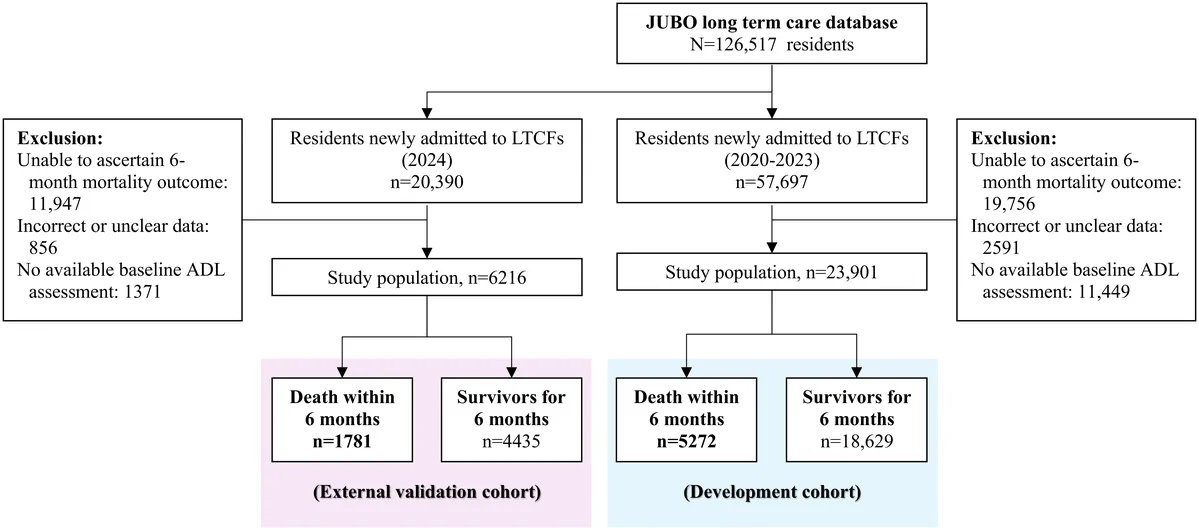
Flow diagram for study population inclusion. Unable to ascertain 6-month mortality outcome: residents discharged or lost to follow-up before completion of the 6-month outcome window whose postdischarge vital status was unavailable in the JUBO database. Residents who died within 6 months while under LTCF follow-up were included as outcome events. Incorrect or unclear data: records with implausible admission dates, inconsistent admission or discharge information, or duplicate records that could not be reconciled. No available baseline ADL assessment: residents with no recorded ADL assessment at admission and therefore no baseline ADL information available for feature construction. Residents with a baseline ADL assessment were retained in the analytic cohort even if individual ADL items were partially missing or if subsequent ADL information was insufficient to derive the ADL-change variable. These values were treated as predictor-level missingness and handled using the prespecified imputation procedure. ADL: activities of daily living; LTCF: long-term care facility.

To be included in this study, residents were not required to remain in the LTCF for the entire 6-month outcome window. Residents who died within 6 months after admission while under LTCF follow-up were classified as outcome events. Residents were excluded if their 6-month vital status could not be reliably ascertained, such as discharge or loss to follow-up before completion of the outcome window without subsequent status information in the JUBO database. The exclusion criteria also included implausible admission dates, duplicate records that could not be reconciled, and absence of any available baseline ADL assessment required to define baseline functional status. The cohort selection process, exclusions, and event counts are reported in Figure 1.

### Outcome, Predictors, and Data Preprocessing

#### Outcome Definition

The primary outcome was all-cause mortality within 6 months after LTCF admission. Mortality status and date of death were ascertained from the discharge or case-closure disposition fields in the JUBO database, which contains the recorded reason for case closure, including death, as documented by facility staff at the time of case closure. The outcome was operationalized as a binary indicator of death occurring within 6 months after LTCF admission. Postdischarge vital status data were not consistently available; thus, residents discharged or lost to follow-up before 6 months without subsequent status information were excluded from the primary analysis.

#### Candidate Predictors

Candidate predictors were prespecified through a 2-step process. First, we conducted a review of published prognostic models and studies on short-term mortality in LTCF and nursing home populations. Several repeatedly reported prognostic domains were identified, including demographics, functional status, comorbidity burden, recent acute care service use, nutritional risk, psychosocial status, and care-related factors (Multimedia Appendix 1). Second, we mapped these domains to the structured variables routinely collected in the JUBO LTCF assessment system, prioritizing predictors that were clinically interpretable, consistently available in routine LTCF workflows, and feasible to implement without linkage to hospital-based electronic health records. Although prior models have incorporated hospital-derived laboratory measures, such information is not consistently available during LTCF admission. Since this study prioritized real-world feasibility, we excluded laboratory variables from the analyses.

In addition to variables available at or shortly after admission, we also considered predictors capturing clinically meaningful changes during the early LTCF stay. This is because the intended use of the model is risk stratification using routinely collected data available at or before the time of risk estimation, not prediction based solely on variables fixed at the moment of admission. We accordingly included selected longitudinal or look-back variables that can be derived from routine LTCF records. Integrating these considerations, we prespecified candidate predictors, including age, sex, recent hospitalization history, level of consciousness measured by the Glasgow Coma Scale [20], ADL score [21], Geriatric Depression Scale-15 score [22], Study of Osteoporotic Fractures score [23], Mini Nutritional Assessment—Short Form [24], Cumulative Illness Rating Scale for Geriatrics score [25], do-not-resuscitate documentation, number of medications, body weight, fall count, denture use, tube feeding, and use of respiratory aids. The selected longitudinal or look-back predictors, including hospitalization history, falls, weight change, and ADL change, were defined using prespecified assessment windows in the LTCF record. The operational definition, data source, and measurement window for each predictor, including whether it was available before admission, at admission, shortly after admission, or during routine follow-up before risk estimation, are detailed in Multimedia Appendix 2.

#### Predictor Measurement and Preprocessing

Predictors were extracted from structured fields recorded at admission and from routine LTCF assessments available before or at the time of risk estimation. Variables recorded after death, or otherwise unavailable at the corresponding risk-estimation time point, were not used.

ADL-related features were handled by distinguishing cohort eligibility from predictor-level missingness. Residents were required to have at least 1 baseline ADL assessment to define baseline functional status; those without such assessment were excluded. Among eligible residents, partially missing ADL items were treated as predictor-level missingness, not exclusion criteria. ADL change was calculated only when at least 2 ADL assessments were available within the prespecified assessment window before risk estimation. Otherwise, ADL change was treated as a missing predictor and handled using the prespecified imputation procedure.

Other variables representing recent clinical instability or longitudinal change, such as hospitalization count, falls, and weight change, were calculated using prespecified look-back or assessment windows before the corresponding risk-estimation time point. Continuous variables were retained on their original scales where appropriate, and categorical variables were encoded using standard binary or categorical representations. To balance data quality with feasibility and interpretability, candidate predictors with high missingness (>30%) were excluded from the model development cohort (Multimedia Appendix 3). The remaining missing values were handled using an imputation strategy.

#### Missing Data Handling

Guided by the clinical documentation patterns of the source database [26-28], we applied a pragmatic rule-based imputation strategy to handle missing values. For binary or count variables typically documented only when present in routine LTCF workflows (eg, do-not-resuscitate documentation, number of hospitalizations within the previous 6 months, falls, use of respiratory assistive devices, tube feeding, and denture use), missing entries were treated as indicating absence (coded as 0). This assumption reflects the usual documentation practice where the event is actively recorded when it occurs, although we acknowledge that under-documentation in some settings may lead to misclassification.

For continuous measures and scale scores, we standardized each variable using *z*-score normalization, with the mean and SD estimated from the model development cohort only. Then, we imputed missing standardized values as 0 (ie, the cohort mean on the original scale). This approach places missing values at a neutral reference point in the distribution, avoiding the imputation of implausible extremes [29]. All preprocessing parameters estimated in the development cohort were applied unchanged to the external validation cohort to prevent information leakage and mirror real-world deployment.

### Model Development, Internal Evaluation, and Calibration

We compared 8 classification algorithms on their predictions of 6-month mortality (Multimedia Appendix 4) to investigate the prognostic advantage of advanced computational techniques over traditional statistical methods. The candidates were categorized into two groups, which are explored hereinafter.

1. Tree-based ensemble learners: This group included an extreme gradient boosting (XGB) classifier (gradient boosting) [30], a random forest classifier (bagging) [31], and our proposed hybrid of extreme gradient boosting and random forest [HybridXGBRF] approach. Ensemble models were selected because of their ability to capture intricate nonlinear feature interactions and robustness against noisy clinical data. The HybridXGBRF model is an ensemble architecture that integrates the predictive strengths of gradient boosting and bagging using a weighted probability blending (soft-voting) mechanism. For each prediction, the model computes a final probability *P*_Hybrid_, which is as described below:

$P_{Hybrid}=\alpha \cdot P_{XGB}+(1-\alpha )\cdot P_{RF},\;\alpha \in [0,1]$

where *P*_XGB_ and *P*_RF_ are the predicted probabilities from the base learners, and α controls their relative contribution. The blending weight (α) was tuned via an exhaustive search over a prespecified range (α∈[0,1]) in the development cohort. For each candidate α, the Hybrid probability was evaluated under the same cross-validation framework. The α yielding the best cross-validated performance (primary criterion: area under the receiver operating characteristic curve [AUROC]) was selected and then fixed for refitting on the full development cohort, as well as evaluation in the external validation cohort.

2. Linear statistical benchmarks: Five linear models were constructed as performance baselines, including standard logistic regression and regularized versions (ie, Ridge, L2 [32]; Lasso, L1 [33]; and Elastic Net [34]), to address potential multicollinearity among clinical predictors.

An internal model evaluation was conducted in the development cohort using 5-fold stratified cross-validation, preserving the event proportion in each fold. Within each fold, all preprocessing steps, including feature screening rules, imputation, standardization (when applicable), and model fitting, were performed using only the training partition and then applied to the corresponding validation partition. This fold-specific pipeline served to minimize information leakage. For each model and fold, we calculated the discrimination and classification performance metrics, as follows: AUROC, accuracy, precision, recall (sensitivity), *F*_1_-score, and confusion matrix. Multimedia Appendix 5 presents the definitions of the evaluation metrics. When the accuracy was summarized across folds, we reported the mean (SD), while probability accuracy was assessed using the Brier score whenever applicable. We prioritized the AUROC as the primary metric for model comparison because it is threshold-independent and less sensitive to class imbalance. Meanwhile, threshold-dependent metrics (precision, recall, or *F*_1_) were reported at the classification threshold used across models for comparability.

Calibration was first evaluated using reliability diagrams. For each model, predicted 6-month mortality risks were grouped into deciles of predicted risk based on the predicted probability distributions. Within each bin, we calculated the mean predicted probability and observed event rate (ie, the proportion of residents who died within 6 months). These values were plotted against the 45-degree line representing perfect calibration. For internal evaluation and to reduce optimism, calibration curves were generated using out-of-fold predictions from the cross-validation procedure; for temporal external validation, calibration curves were generated using predictions from the final model applied to the 2024 cohort.

In addition to graphical and decile-based calibration assessments, we calculated calibration intercept, calibration slope, observed-to-expected ratio, and Brier score for the final HybridXGBRF model in both cohorts. Calibration intercept and slope were estimated using logistic recalibration; the observed-to-expected ratio was calculated as observed deaths divided by the sum of predicted probabilities; and the Brier score as the mean square difference between observed outcomes and predicted probabilities. CIs were estimated using resident-level bootstrap resampling. We also summarized calibration for the final HybridXGBRF model using risk-decile calibration tables, reporting the number of residents, observed deaths, observed risk, mean predicted risk, and predicted-risk range within each decile.

### Temporal External Validation, Calibration, and Subgroup Analyses

To assess generalizability under a temporal dataset shift, we performed temporal external validation in the 2024 cohort (admissions in 2024), which was not used for model development, feature engineering decisions, or cross-validation. The final models were refitted to the full development cohort using the same predictors and preprocessing rules, and then evaluated in the external validation cohort.

All preprocessing parameters estimated in the development cohort (eg, scaling parameters for continuous variables) were applied to the external validation cohort. Model performance in the external validation was quantified using the same set of metrics as in the internal evaluation (Multimedia Appendix 5).

We additionally evaluated discrimination across sex, age (>85 and ≤85 years), and ADL decline (yes or no) to examine performance stability, reporting subgroup AUROC values using the same prediction pipeline.

### Clinical Utility and Threshold Analyses

We conducted 2 complementary analyses for the HybridXGBRF model in the temporal external validation cohort in order to assess potential clinical utility beyond discrimination. First, we performed decision-curve analysis: net benefit was calculated across a range of threshold probabilities and compared with default strategies of classifying all residents as high risk or no residents as high risk. Second, we evaluated threshold-specific performance using clinically interpretable predicted-risk thresholds: for each threshold, we reported sensitivity (recall), specificity, positive predictive value, negative predictive value, *F*_1_-score, and the proportion of residents classified as high risk. This analysis was intended to examine the trade-offs between case detection and alert burden under alternative risk-stratification thresholds.

### Model Interpretability and Individual-level Risk Explanation

In an attempt to enhance clinical interpretability, we quantified feature contributions using Shapley Additive Explanations (SHAP) [35,36]. Because HybridXGBRF generates the final predicted probability as a weighted blend of the XGB and random forest component predictions, SHAP values were computed for the final HybridXGBRF blended prediction function. The reported feature attributions correspond to the final predicted probability of 6-month mortality, not to either component model alone. Global interpretability was visualized using SHAP beeswarm, and forest plots were used to rank feature importance across the study population. Local interpretability for individual-level risk explanations was demonstrated using SHAP force plots, illustrating how specific clinical features shifted the final HybridXGBRF-predicted probability from the baseline risk estimate.

### Statistical Analysis

The descriptive characteristics of residents in the development cohort were summarized overall and by the 6-month mortality status. Group differences were quantified using standardized mean differences to describe the magnitude of imbalance between residents who died within 6 months and those who survived.

Intending to assess potential selection bias regarding cohort exclusion, we compared baseline characteristics between the development cohort and 2 major excluded groups: residents excluded because their 6-month mortality outcome could not be reliably ascertained and residents excluded because no baseline ADL assessment was available.

Model performance metrics were computed for internal evaluation and temporal external validation, as described earlier. For AUROC, precision, recall, *F*_1_-score, accuracy, Brier score, and subgroup-specific performance metrics, 95% CIs were calculated using resident-level bootstrap resampling of the evaluation set.

For the purpose of comparing the discriminative performance of HybridXGBRF with XGB, we conducted paired bootstrap comparisons of AUROC within each cohort using 2000 resident-level bootstrap resamples. In each bootstrap sample, AUROC was calculated for both models using the same resampled residents, and the paired AUROC difference was computed as AUROC_HybridXGBRF−AUROC_XGB. The 95% CI was estimated from the empirical distribution of the paired AUROC differences, and 2-sided *P* values were calculated to test whether the paired difference differed from 0. Statistical significance was set at *P*<.05. Database management and extraction were conducted using PostgreSQL, and all statistical analyses were performed using Python (version 3.12.7; Python Software Foundation).

### Sensitivity Analyses

The primary binary 6-month mortality evaluation was complemented by a time-to-event discrimination analysis using cumulative or dynamic area under the curve (AUC) at monthly time points before the 6-month end point. At each time point, cases were defined as residents who had died by that time, and controls were defined as residents who remained event-free beyond that time. Cumulative or dynamic AUC was calculated separately for the development and temporal external validation cohorts. This analysis evaluated whether model discrimination remained stable across earlier time horizons, while the primary model objective remained 6-month mortality risk prediction.

A concern arises here that missing binary or count variables may represent incomplete documentation, not true clinical absence. Thus, 2 additional post-hoc analyses were conducted to tackle this issue. For one, we examined whether missingness in selected key clinical assessments, including subsequent ADL reassessment, initial body weight, and Glasgow Coma Scale score, was associated with 6-month mortality by using missingness indicators for these variables as exposures in adjusted logistic regression models. For another, facility-level variation in overall documentation missingness was examined while stratified by facility size and geographic region, and heterogeneity across individual facilities was tested using a global chi-square test of independence. This analysis was conducted to assess whether missingness patterns reflected institutional documentation practices in addition to patient-level clinical status.

### Ethical Considerations

This study was approved by the institutional review board of National Cheng Kung University Hospital (B-ER-112‐466). All data were anonymized prior to analysis, and the use of AI models was aligned with the ethical principles of transparency, clinical utility, and human oversight.

## Results

### Study Population and Characteristics

Between 2020 and 2023, 57,697 newly admitted LTCF residents were identified in the development cohort. After excluding residents whose 6-month mortality outcome could not be reliably identified (n=19,756), records with incorrect or unclear data (n=2591), and residents with no available baseline ADL assessment required for feature construction (n=11,449), the final development cohort included 23,901 residents from 493 LTCFs. Among them, 5272 residents died within 6 months after admission and 18,629 survived for at least 6 months. The participant flow diagram is presented in Figure 1.

Residents discharged or lost to follow-up before completion of the 6-month outcome window with unknown subsequent vital status were compared with the analytic cohort (Table S1 in Multimedia Appendix 6). Compared with the analytic cohort, this excluded group was younger and had higher ADL scores, higher body weight, lower prevalence of do-not-resuscitate documentation, and a higher prevalence of denture use. Facility size was similar between groups, although facility region differed; indicators of greater medical dependency, including tube feeding and respiratory support were not significantly different.

Residents excluded due to the absence of a baseline ADL assessment were similarly compared with the analytic cohort (Table S2 in Multimedia Appendix 6). This excluded group (vs analytic cohort) was slightly younger and had lower Modified Cumulative Illness Rating Scale for Geriatric Patients scores, lower prevalence of do-not-resuscitate documentation, fewer hospital admissions within 6 months, and markedly lower documentation of denture use, tube feeding, respiratory support, and fall history.

The characteristics of the development cohort are summarized in Table 1. The mean age was 79.3 (SD 11.8) years, and 12,155 (50.9%) residents were female. Residents who died within 6 months (vs who survived for at least 6 months) had greater functional impairment, lower body weight, more frequent hospital admissions, and higher use of tube feeding and respiratory support. Standardized mean differences greater than 0.1 were observed for Cumulative Illness Rating Scale for Geriatrics, ADL measures, body weight, number of hospitalizations, sex, do-not-resuscitate documentation, denture use, tube feeding, respiratory support, and institutional region.

**Table 1.** Characteristics of the development cohort (n=23,901).

Characteristic	Total (n=23,901)	Death group (n=5272)	Survivor group (n=18,629)	SMD^a^
Age (years), mean (SD)	79.3 (11.8)	80.2 (11.5)	79.1 (11.9)	0.09
Age group (years), n (%)				0.06
<65	2542 (10.6)	521 (9.9)	2021 (10.8)	
65‐69	1926 (8.1)	430 (8.2)	1496 (8)	
70‐74	2794 (11.7)	549 (10.4)	2245 (12.1)	
75‐79	3112 (13)	623 (11.8)	2489 (13.4)	
80‐84	4345 (18.2)	1001 (19)	3344 (18)	
85‐89	4734 (19.8)	1075 (20.4)	3659 (19.6)	
90+	4448 (18.6)	1073 (20.4)	3375 (18.1)	
Sex, n (%)				0.23
Male	11,746 (49.1)	3069 (58.2)	8677 (46.6)	
Female	12,155 (50.9)	2203 (41.8)	9952 (53.4)	
CIRS-G^b^, mean (SD)	1.7 (0.6)	1.8 (0.6)	1.7 (0.6)	0.17
DNR^c^ notes (+), n (%)	9777 (40.9)	2472 (46.9)	7305 (39.2)	0.15
ADL^d^, mean (SD)				
First score	28.6 (31.7)	15.1 (22.9)	32.3 (32.8)	−0.61
Maximum score in 6 months	31 (32.8)	15.4 (23.3)	35.4 (33.7)	−0.69
Last score	28.5 (31.8)	13.3 (21.7)	30.5 (32.4)	−0.63
GCS^e^ (EVM), mean (SD)^f^	7.7 (5.3)	7.0 (4.9)	7.9 (5.4)	−0.16
Dentures, n (%)	3918 (16.4)	720 (13.7)	3198 (17.2)	−0.13
Tube feeding, n (%)	2081 (8.7)	758 (14.4)	1323 (7.1)	0.27
Respiratory support, n (%)	848 (3.5)	410 (7.8)	438 (2.4)	0.26
History of falls, n (%)	1197 (5)	294 (5.6)	903 (4.8)	0.03
BW^g^, mean (SD)	51.4 (12.1)	49.1 (13.8)	52 (11.5)	−0.22
Number of admissions in 6 months, mean (SD)	0.7 (1.1)	1.1 (1.1)	0.5 (1)	0.53
Institutional regions, n (%)				0.14
North	2245 (9.4)	378 (7.2)	1867 (10)	
Central	4754 (19.9)	1122 (21.3)	3632 (19.5)	
South	16,603 (69.5)	3745 (71)	12,861 (69)	
East	299 (1.3)	30 (0.6)	269 (1.4)	

a SMD: standardized mean difference.

b CIRS-G: Modified Cumulative Illness Rating Scale for Geriatric Patients.

c DNR: do not resuscitate.

d ADL: activities of daily living.

e GCS: Glasgow Coma Scale.

f GCS scores between 3 and 15. It comprises 3 parameters: best eye response (E), best verbal response (V), and best motor response (M).

g BW: body weight.

In 2024, a total of 20,390 newly admitted LTCF residents were identified for temporal external validation. After applying the predefined exclusion criteria, the temporal external validation cohort included 6216 residents from 548 LTCFs. Among them, 1781 residents died within 6 months after admission, and 4435 survived for at least 6 months. The mean age was 78.6 (SD 11.7) years, and 3043 (49%) residents were female. Baseline characteristics of the temporal external validation cohort are presented in Table 2.

**Table 2.** Characteristics of the external validation cohort (n=6216).

Characteristic	Total (n=6216)	Death group (n=1781)	Survivor group (n=4435)
Age (years), mean (SD)	78.6 (11.7)	79.5 (11.4)	78.2 (11.9)
Age group (years), n (%)			
<65	711 (11.4)	183 (10.3)	528 (11.9)
65‐69	531 (8.5)	143 (8)	388 (8.7)
70‐74	786 (12.6)	230 (12.9)	556 (12.5)
75‐79	864 (13.9)	212 (11.9)	652 (14.7)
80‐84	1116 (18)	325 (18.2)	791 (17.8)
85‐89	1388 (22.3)	415 (23.3)	973 (21.9)
90+	820 (13.2)	273 (15.3)	547 (12.3)
Sex, n (%)			
Male	3173 (51)	1067 (59.9)	2106 (47.5)
Female	3043 (49)	714 (40.1)	2329 (52.5)
DNR^a^ notes (+), n (%)	2832 (45.6)	946 (53.1)	1886 (42.5)
ADL^b^, mean (SD)			
First score	26.3 (30.1)	15 (23.2)	30.7 (31.3)
Maximum score in 6 months	28.9 (31.2)	15.3 (23.5)	34.4 (32.2)
Last score	26.6 (30)	12.2 (21)	29.1 (30.7)
GCS^c^ (EVM), mean (SD)^d^	7.9 (5.4)	7(5)	8.2 (5.5)
Dentures, n (%)	783 (12.6)	151 (8.5)	632 (14.3)
Tube feeding, n (%)	888 (14.3)	378 (21.2)	510 (11.5)
Respiratory support, n (%)	427 (6.9)	249 (14)	178 (4)
History of falls, n (%)	618 (9.9)	191 (10.7)	427 (9.6)
BW^e^, mean (SD)	52.7 (11.8)	50.5 (12)	53.5 (11.7)
Number of admissions in 6 months, mean (SD)	0.7 (1.1)	1 (1.1)	0.6 (1.1)
Institutional regions, n (%)			
North	462 (7.4)	138 (7.7)	324 (7.3)
Central	1328 (21.4)	407 (22.9)	921 (20.8)
South	4333 (69.7)	1217 (68.3)	3116 (70.3)
East	93 (1.5)	19 (1.1)	74 (1.7)

a DNR: do not resuscitate.

b ADL: activities of daily living.

c GCS: Glasgow Coma Scale.

d GCS scores between 3 and 15. It comprises 3 parameters: best eye response (E), best verbal response (V), and best motor response (M).

e BW: body weight.

### Model Development, Internal Evaluation, and Calibration

**Figure 2.**
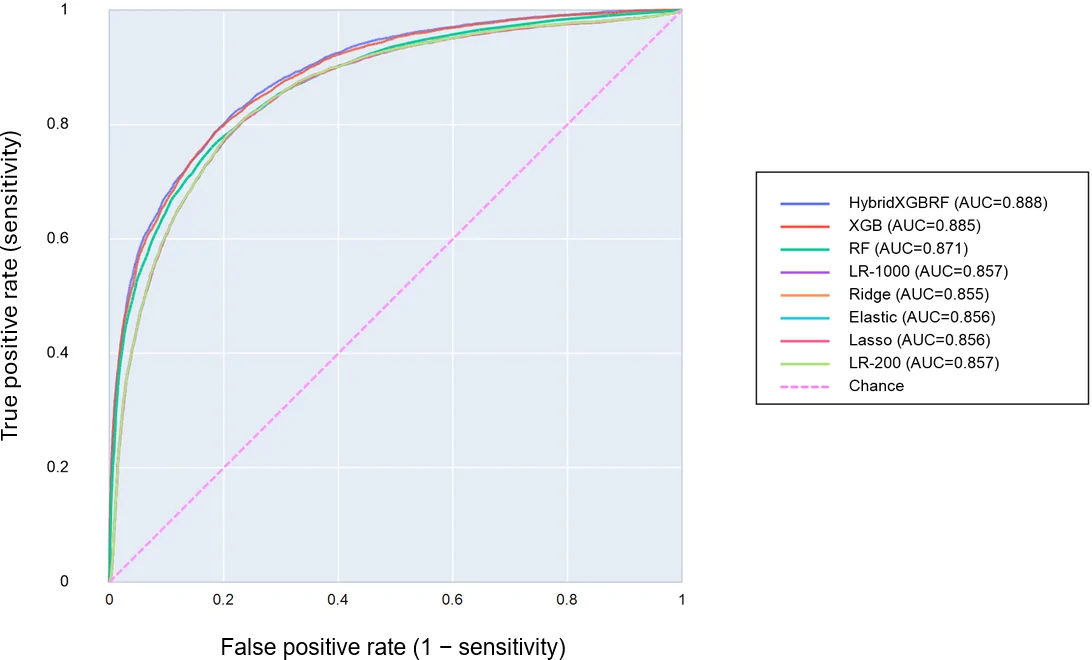
Receiver operating characteristic curve comparison. AUC: area under the curve; HybridXGBRF: hybrid of extreme gradient boosting and random forest; LR: logistic regression; RF: random forest; XGB: extreme gradient boosting.

The performance of the 8 classification algorithms in the internal evaluation is summarized in Table 3 and Figure 2. Overall, the linear models were outperformed by the tree-based models (eg, HybridXGBRF, XGB, and random forest), achieving higher AUROC values (0.87‐0.89 vs 0.86), higher sensitivity or recall (0.51‐0.54 vs 0.48‐0.49), and higher *F*_1_-scores (0.62‐0.64 vs 0.57‐0.58).

**Table 3.** Performance of the different predictive models.

Model metrics	Model 1^a^	Model 2^b^	Model 3^c^	Model 4^d^	Model 5^e^	Model 6^f^	Model 7^g^	Model 8^h^
AUROC^i^ (95% CI)	*0.89 (0.88‐0.89)* ^j^	0.88 (0.88‐0.89)	0.87 (0.87‐0.88)	0.86 (0.85‐0.86)	0.86 (0.85‐0.86)	0.86 (0.85‐0.86)	0.86 (0.85‐0.86)	0.86 (0.85‐0.86)
Precision (95% CI)	0.79 (0.78‐0.80)	*0.79 (0.78‐0.80)*	0.75 (0.74‐0.76)	0.70 (0.69‐0.71)	0.70 (0.69‐0.72)	0.70 (0.69‐0.71)	0.70 (0.69‐0.71)	0.70 (0.69‐0.71)
Sensitivity or recall (95% CI)	0.54 (0.52‐0.55)	0.51 (0.50‐0.52)	*0.54 (0.52‐0.55)*	0.49 (0.48‐0.51)	0.48 (0.47‐0.50)	0.48 (0.47‐0.50)	0.49 (0.47‐0.50)	0.49 (0.48‐0.51)
Specificity	0.96	0.96	0.95	0.94	0.94	0.94	0.94	0.94
*F*_1_-score (95% CI)	*0.64 (0.63‐0.65)*	0.62 (0.61‐0.63)	0.62 (0.61‐0.64)	0.58 (0.56‐0.59)	0.57 (0.56‐0.59)	0.57 (0.56‐0.59)	0.57 (0.56‐0.59)	0.58 (0.56‐0.59)
Accuracy (95% CI)	*0.87 (0.86‐0.87)*	0.86 (0.86‐0.87)	0.86 (0.85‐0.86)	0.84 (0.84‐0.84)	0.84 (0.84‐0.85)	0.84 (0.84‐0.85)	0.84 (0.84‐0.85)	0.84 (0.84‐0.84)
Brier score (95% CI)	*0.10 (0.10‐0.10)*	0.10 (0.10‐0.11)	0.11 (0.10‐0.11)	0.12 (0.11‐0.12)	0.12 (0.11‐0.12)	0.12 (0.11‐0.12)	0.12 (0.11‐0.12)	0.12 (0.11‐0.12)

a HybridXGBRF (hybrid of extreme gradient boosting and random forest; proposed approach).

b Extreme gradient boosting classifier.

c Random forest classifier.

d Logistic regression (max-iter =1000).

e Ridge.

f Elastic.

g Lasso.

h Logistic regression (max-iter =200).

i AUROC: area under the receiver operating characteristic curve.

j The italics format is used to highlight the best values.

Among all evaluated algorithms, the HybridXGBRF model achieved the best overall internal performance, with the highest AUROC (0.89, 95% CI 0.88‐0.89), highest *F*_1_-score (0.64, 95% CI 0.63‐0.65), highest accuracy (0.87, 95% CI 0.86‐0.87), and lowest Brier score (0.10, 95% CI 0.10‐0.10). The linear models contrasted this, showing lower classification performance, particularly for sensitivity and *F*_1_-score.

**Figure 3.**
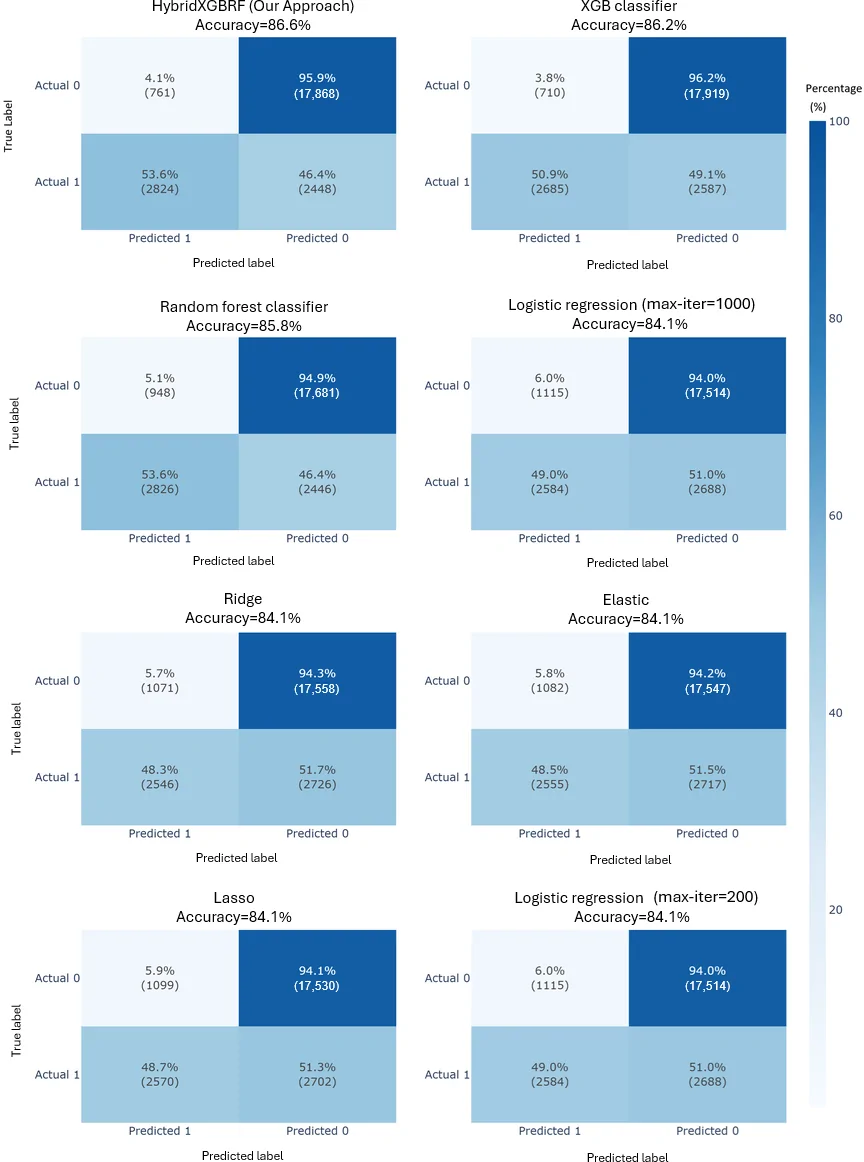
Confusion matrix comparisons between machine learning classifiers. HybridXGBRF: hybrid of extreme gradient boosting and random forest; XGB: extreme gradient boosting.

Figure 3 presents the confusion matrices of the evaluated models. Specificity remained consistently high across models (range 0.94‐0.96), and sensitivity was more modest (range 0.48‐0.54). Compared with XGB, the HybridXGBRF model identified more positive cases (2824 vs 2685) and achieved higher sensitivity and *F*_1_-score, although XGB generated fewer false-positive predictions (710 vs 761). These findings are aligned with the AUROC, *F*_1_-score, and Brier score results reported in Table 3.

**Figure 4.**
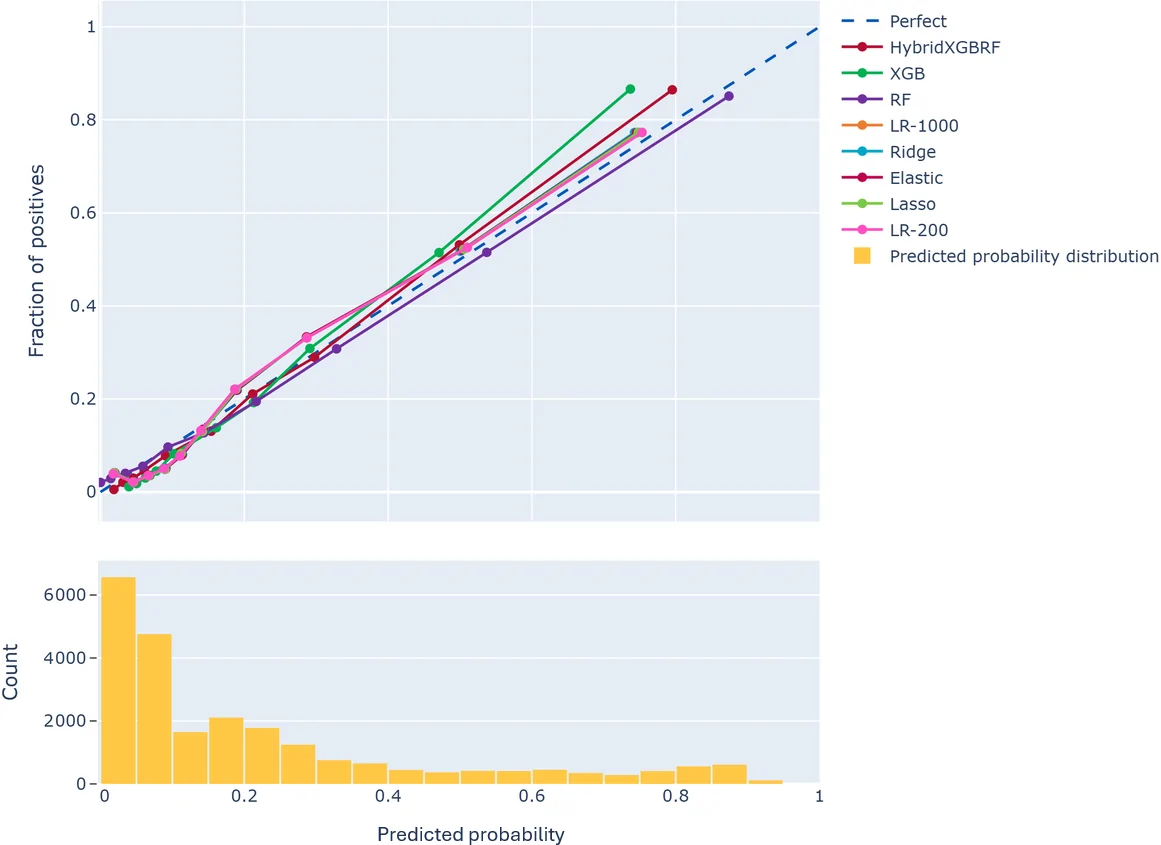
Calibration plots and distributions of the machine learning models in the development cohort. HybridXGBRF: hybrid of extreme gradient boosting and random forest; LR: logistic regression; RF: random forest; XGB: extreme gradient boosting.

The HybridXGBRF model also demonstrated strong internal calibration. The observed-to-expected (O/E) ratio was 1.00 (Multimedia Appendix 7). Predicted probabilities closely followed the reference 45-degree line across the low-to-moderate predicted-risk range (Figure 4) based on the risk-decile distribution (Table S1 in Multimedia Appendix 8). A small degree of underestimation was observed in the highest predicted-risk bin (>0.7), while quantitative calibration metrics showed a calibration slope of 1.21 and an intercept of 0.20. Overall, the HybridXGBRF model achieved the lowest internal Brier score (0.10) among the evaluated models (Multimedia Appendix 9), and tree-based models generally showed lower Brier scores than linear models (Table 3). In paired bootstrap comparisons, HybridXGBRF demonstrated a statistically significant but small AUROC improvement over XGB (Multimedia Appendix 10). In the development cohort, the AUROC difference was 0.003 (95% CI 0.002‐0.005; *P*<.001).

### Temporal External Validation, Calibration, and Subgroup Analyses

In the temporal external validation cohort, the models’ predictive performance remained stable, indicating good temporal generalizability. The HybridXGBRF model achieved an AUROC of 0.90 (95% CI 0.89‐0.91), comparable to its internal evaluation performance, as well as achieved an *F*_1_-score of 0.68 (95% CI 0.66‐0.70), accuracy of 0.85 (95% CI 0.84‐0.86), and Brier score of 0.11 (95% CI 0.11‐0.12; Table 4).

**Table 4.** Performance metrics of classification algorithms in the external validation dataset^a^.

Model metrics	Model 1^b^	Model 2^c^	Model 3^d^	Model 4^e^	Model 5^f^	Model 6^g^	Model 7^h^	Model 8^i^
AUROC^k^ (95% CI)	*0.90 (0.89‐0.91)*	0.89 (0.89‐0.90)	0.88 (0.87‐0.89)	0.87 (0.86‐0.88)	0.87 (0.86‐0.88)	0.87 (0.86‐0.88)	0.87 (0.86‐0.88)	0.87 (0.86‐0.88)
Precision (95% CI)	*0.86 (0.84‐0.88)*	*0.86 (0.84‐0.88)*	0.81 (0.79‐0.83)	0.77 (0.75‐0.79)	0.78 (0.75‐0.80)	0.78 (0.75‐0.80)	0.77 (0.75‐0.80)	0.77 (0.75‐0.79)
Sensitivity or recall (95% CI)	*0.57 (0.55‐0.59)*	0.54 (0.52‐0.57)	0.56 (0.54‐0.59)	0.54 (0.51‐0.56)	0.53 (0.51‐0.55)	0.53 (0.51‐0.55)	0.53 (0.51‐0.55)	0.54 (0.51‐0.56)
*F*_1_-score (95% CI)	*0.68 (0.66‐0.70)*	0.67 (0.65‐0.68)	0.67 (0.65‐0.68)	0.63 (0.61‐0.65)	0.63 (0.61‐0.65)	0.63 (0.61‐0.65)	0.63 (0.61‐0.65)	0.63 (0.61‐0.65)
Accuracy (95% CI)	*0.85 (0.84‐0.86)*	0.84 (0.84‐0.85)	0.84 (0.83‐0.85)	0.82 (0.81‐0.83)	0.82 (0.81‐0.83)	0.82 (0.81‐0.83)	0.82 (0.81‐0.83)	0.82 (0.81‐0.83)
Brier score (95% CI)	*0.11 (0.11‐0.12)*	0.12 (0.11‐0.12)	0.12 (0.11‐0.12)	0.13 (0.12‐0.13)	0.13 (0.13‐0.13)	0.13 (0.13‐0.13)	0.13 (0.12‐0.13)	0.13 (0.12‐0.13)

a Italicized values are the best ones among the evaluated models.

b HybridXGBRF (hybrid of extreme gradient boosting and random forest; proposed approach).

c Extreme gradient boosting classifier.

d Random forest classifier.

e Logistic regression (max-iter=1000).

f Ridge.

g Elastic.

h Lasso.

i Logistic regression (max-iter=200).

j AUROC: area under the receiver operating characteristic curve.

Tree-based models again outperformed linear models in temporal external validation. Tree-based models (vs linear models) showed higher AUROC values (0.88‐0.90 vs 0.87), higher *F*_1_-scores (0.67‐0.68 vs 0.63), and higher accuracies (0.84‐0.85 vs 0.82). In the paired bootstrap comparison, HybridXGBRF showed a small but statistically significant AUROC improvement over XGB in the temporal external validation cohort, with an AUROC difference of 0.004 (95% CI 0.001‐0.006; *P*=.003; Multimedia Appendix 10).

**Figure 5.**
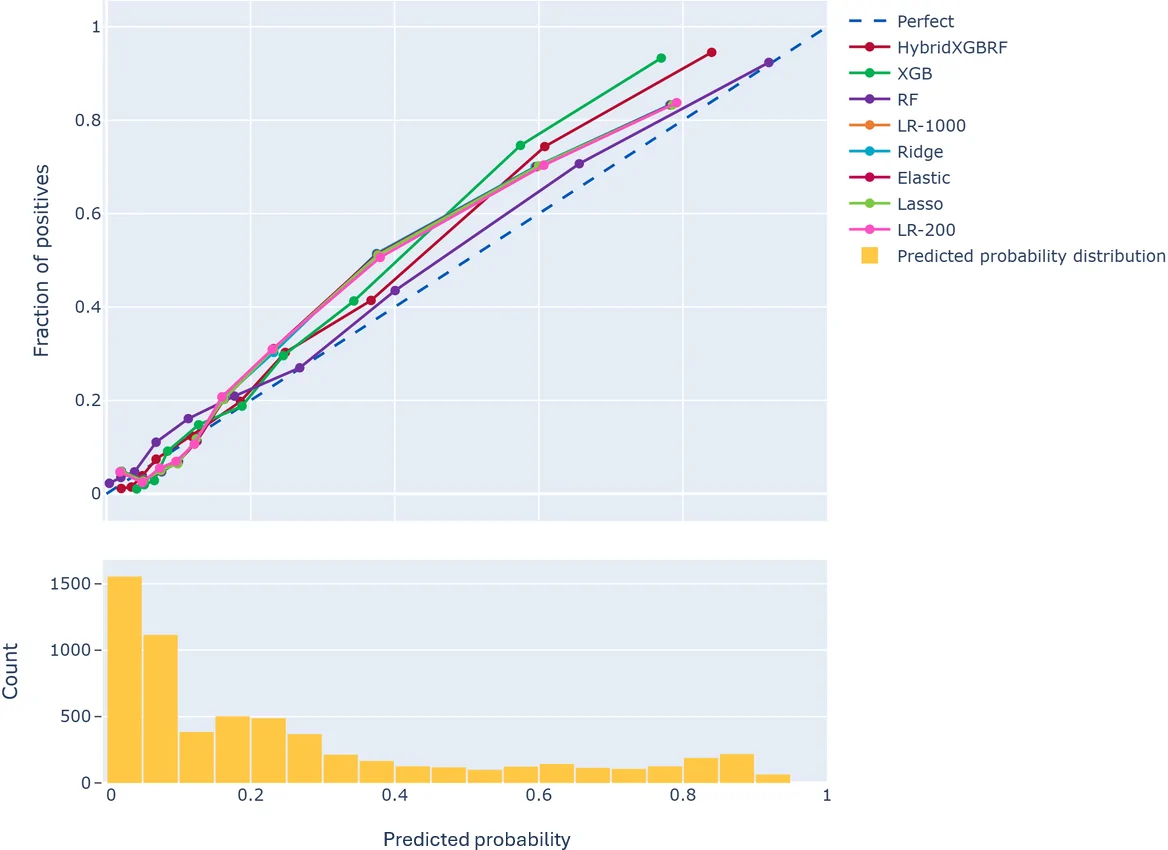
Calibration plots and distributions of the machine learning models in the external validation cohort. HybridXGBRF: hybrid of extreme gradient boosting and random forest; LR: logistic regression; RF: random forest; XGB: extreme gradient boosting.

External validation showed that HybridXGBRF maintained the strongest calibration among the evaluated models, with the lowest Brier score (0.11; Figure 5 and Table 4). Regarding quantitative calibration metrics, the O/E ratio was 1.13, calibration slope of 1.27, and intercept of 0.52 (Multimedia Appendix 7). The detailed risk-decile analysis (Table S2 in Multimedia Appendix 8) showed that the calibration curve closely followed the reference line across most probability ranges, with mild divergence at higher predicted risks. Tree-based models demonstrated better calibration robustness than linear models (Multimedia Appendix 9).

Subgroup analyses demonstrated that the HybridXGBRF model maintained high and consistent discrimination across sex and age strata, with AUROC values ranging from 0.89 to 0.90 (Table 5). It also showed high discrimination among residents with ADL improvement, with an AUROC of 0.91 (95% CI 0.90‐0.92), *F*_1_-score of 0.71 (95% CI 0.69‐0.73), and accuracy of 0.85 (95% CI 0.84‐0.86). Performance was lower among residents with ADL decline, with an AUROC of 0.71 (95% CI 0.66‐0.75) and sensitivity of 0.03 (95% CI 0.01‐0.07).

**Table 5.** Subgroup performance of the HybridXGBRF^a^ model^b^.

Subgroup	AUROC^c^ (95% CI)	Precision (95% CI)	Sensitivity or recall (95% CI)	*F*_1_-score (95% CI)	Accuracy (95% CI)	Support, n	Positives, n
ADL^d^ improvement	*0.91 (0.90‐0.92)*	0.86 (0.84‐0.87)	0.61 (0.58‐0.63)	0.71 (0.69‐0.73)	0.85 (0.84‐0.86)	5524	1658
ADL decline	0.71 (0.66‐0.75)	0.67 (0.25‐1.00)	0.03 (0.01‐0.07)	0.06 (0.02‐0.12)	0.83 (0.82‐0.83)	692	123
Age >85 years	*0.90 (0.89‐0.92)*	0.87 (0.84‐0.90)	0.60 (0.57‐0.64)	0.71 (0.68‐0.74)	0.85 (0.83‐0.86)	1924	600
Age ≤85 years	0.90 (0.89‐0.91)	0.85 (0.83‐0.87)	0.55 (0.52‐0.58)	0.67 (0.64‐0.69)	0.85 (0.84‐0.86)	4292	1181
Female	*0.90 (0.89‐0.91)*	0.84 (0.80‐0.87)	0.54 (0.50‐0.57)	0.65 (0.62‐0.68)	0.87 (0.86‐0.88)	3043	714
Male	0.89 (0.88‐0.91)	0.87 (0.84‐0.89)	0.59 (0.56‐0.62)	0.70 (0.68‐0.72)	0.83 (0.82‐0.84)	3173	1067

a HybridXGBRF: hybrid of extreme gradient boosting and random forest.

b Italicized values are the best ones among the evaluated models.

c AUROC: area under the receiver operating characteristic curve.

d ADL: activities of daily living.

### Clinical Utility and Threshold Analyses

Decision-curve analysis showed that the HybridXGBRF model provided higher net benefit than the default strategies. These default strategies involved classifying all residents as high risk or no residents as high risk across a clinically relevant range of threshold probabilities in the temporal external validation cohort (Multimedia Appendix 11).

Alternative threshold analyses demonstrated the expected trade-off between case detection and alert burden (Multimedia Appendix 12). At a predicted-risk threshold of 0.20, the model achieved high sensitivity (0.86) and negative predictive value (0.93), but classified 2659 residents as high risk, corresponding to 42.8% of the temporal external validation cohort. At a threshold of 0.30, the model provided a more balanced profile, with sensitivity of 0.72, specificity of 0.88, positive predictive value of 0.71, and the highest *F*_1_-score among the evaluated thresholds (0.72). At a threshold of 0.50, specificity and positive predictive value increased to 0.961 and 0.86, respectively, but sensitivity decreased to 0.57. The combination of the findings here outlines that lower thresholds prioritized sensitivity at the cost of more high-risk classifications, whereas higher thresholds prioritized specificity and positive predictive value at the cost of reduced sensitivity.

### Model Interpretability and Individual-Level Risk Explanation

**Figure 6.**
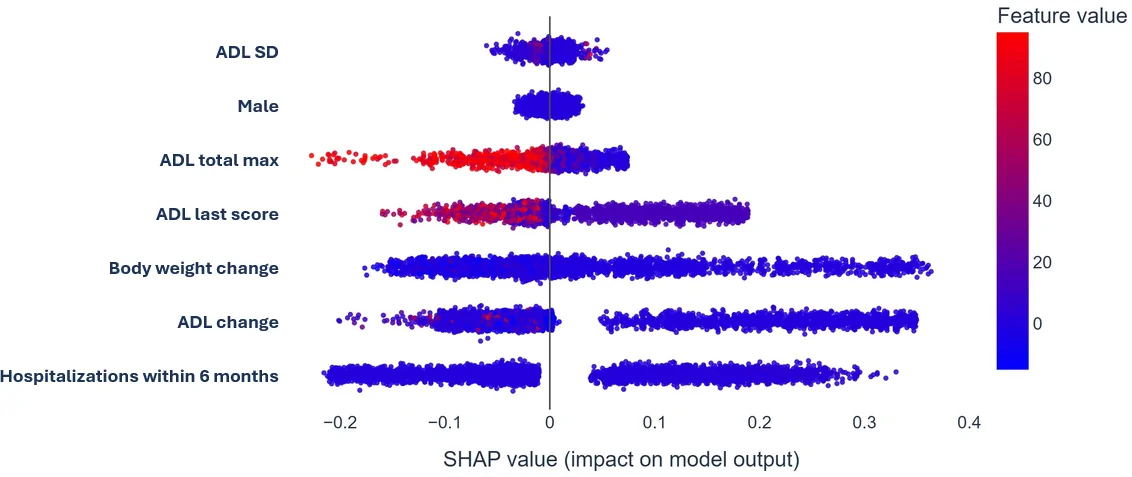
SHAP beeswarm plot of the HybridXGBRF model. ADL: activities of daily living; HybridXGBRF: hybrid of extreme gradient boosting and random forest; SHAP: Shapley Additive Explanations.

**Figure 7.**
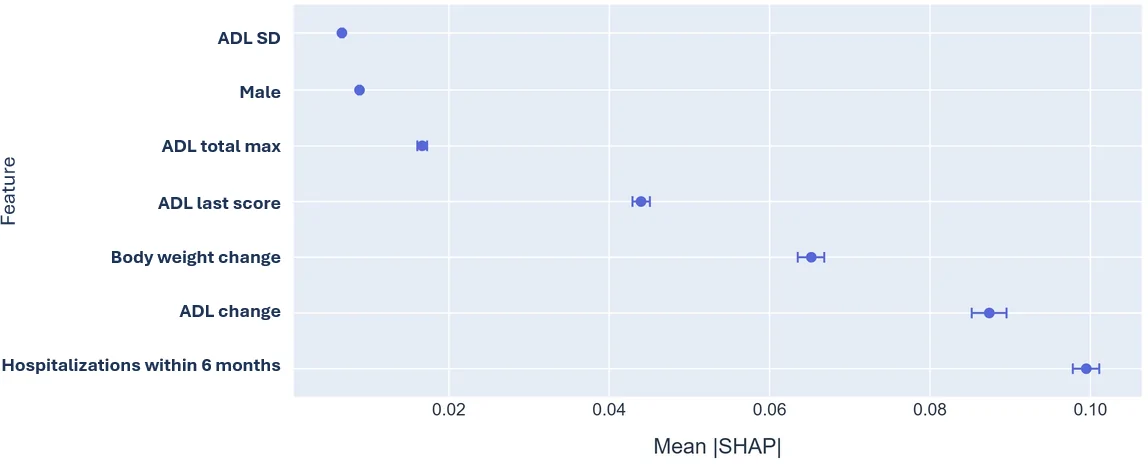
SHAP forest plot of the HybridXGBRF model. ADL: activities of daily living; HybridXGBRF: hybrid of extreme gradient boosting and random forest; SHAP: Shapley Additive Explanations.

Figure 6 (ie, showing the distribution of individual SHAP values in a beeswarm plot) and Figure 7 (ie, showing the mean absolute SHAP values with 95% CIs in a forest plot) present SHAP-based explanations for the final HybridXGBRF blended prediction output. Across both visualizations, recent hospitalizations within the past 6 months made the largest overall contribution to the final HybridXGBRF-predicted 6-month mortality risk, with higher hospitalization counts generally contributing positively to predicted risk.

Functional status was also a key determinant of predicted mortality risk. More recent and lower ADL scores, ADL decline, and variability across ADL measurements were associated with increased predicted risk, as reflected by the predominantly positive SHAP values. Conversely, higher maximum ADL scores contributed modestly to lower predicted mortality risk.

Nutritional indicators were similarly influential. Sequential declines in body weight shifted SHAP values upward, indicating heightened predicted risk following nutritional deterioration. Male residents generally showed slightly higher predicted mortality risk than female residents, hence showing that sex contributed to the prediction.

**Figure 8.**
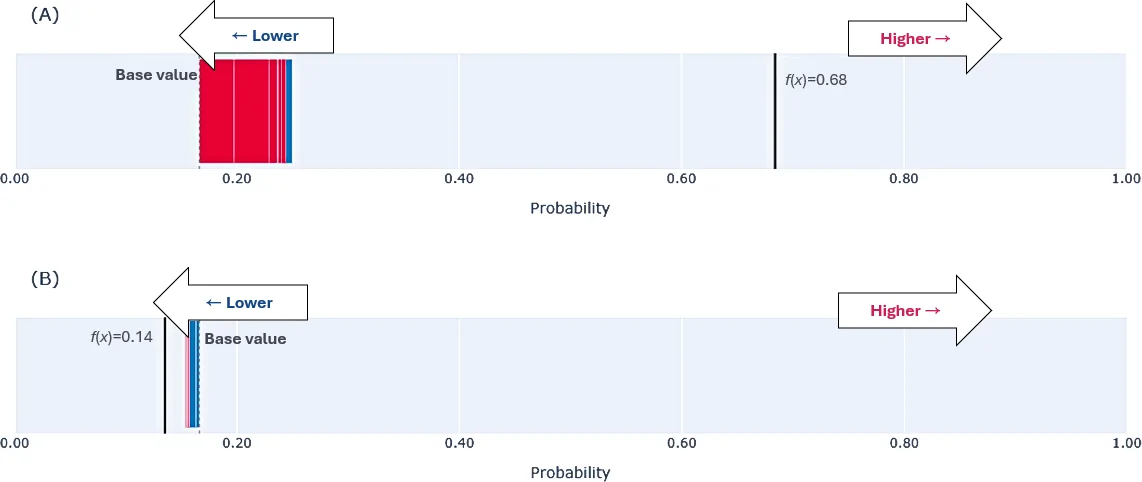
SHAP force plots for 2 representative residents. (A) Positively predicted patient and (B) negatively predicted patient. SHAP: Shapley Additive Explanations.

Figure 8 illustrates the SHAP force plots for 2 representative residents. For the positively predicted case (Figure 8A), multiple high-risk features collectively shifted the prediction from the baseline mortality probability (~0.21) to a substantially higher estimated risk (0.68). The contributing factors (ie, pushed the prediction to a higher risk) included impaired functional status, recent functional decline, lower or decreasing body weight, male sex, and recent hospitalization. For the negatively predicted case (Figure 8B), a minimal deviation from the baseline probability (~0.14) was observed, with predominantly protective features (eg, preserved functional ability, stable body weight, and the absence of recent acute care service use) driving the SHAP values toward a lower predicted risk. These examples highlight the manner in which the HybridXGBRF model integrates functional, nutritional, and clinical history features to generate patient-specific risk estimates.

### Sensitivity Analyses

In both cohorts, cumulative or dynamic AUCs remained above 0.89 across months 1 to 5, indicating stable discrimination across the earlier time horizons (Multimedia Appendix 13). In the sensitivity analysis evaluating missingness mechanisms, missingness in subsequent ADL assessment, initial body weight, and Glasgow Coma Scale score was significantly associated with 6-month mortality in both cohorts (adjusted odds ratio ranges 13.7‐16.6, 3.0‐3.1, and 1.2‐1.3, respectively; all *P*≤.01; Multimedia Appendix 14). Overall documentation missingness varied significantly across the 493 individual facilities (*χ*^2^_492_=140,786.0; *P*<.001; Cramer *V*=0.451), with minimal variation by facility size (7.7%‐8.0%) and greater variation by region (5.5%‐12.1%; Multimedia Appendix 15)[37].

## Discussion

### Principal Findings

This study developed and temporally externally validated an explainable machine learning model for predicting 6-month all-cause mortality among newly admitted LTCF residents using routinely collected LTCF assessment data. In the temporal external validation cohort, the HybridXGBRF model achieved strong discrimination (ie, AUROC of 0.90) without requiring integration with hospital electronic health records nor data collection beyond standard LTCF assessments. Although HybridXGBRF achieved the highest AUROC among the evaluated models, the absolute improvement over XGB was modest. SHAP analysis pinpointed the most influential predictors as frequent hospitalizations within 6 months, ADL impairment, and weight loss, providing clinically interpretable risk attributions at the individual level. The model also maintained stable discrimination across the sex and age subgroups. Therefore, a parsimonious set of routinely collected LTCF variables, when analyzed through an interpretable machine learning framework, may support risk stratification and structured care planning in Taiwanese long-term care settings.

### Interpretation in Context of the Existing Literature

The performance of our model compares favorably with that of established prognostic tools for LTCF populations. The MDS Mortality Risk Index is a validated prognostic tool designed to estimate the risk of 6-month mortality in nursing home residents, and it uses routinely collected clinical and demographic data from the MDS assessment [5,6,8]. The original MDS Mortality Risk Index and its revised versions (MDS Mortality Risk Index-Revised and MDS Mortality Risk Index [version 3]) have demonstrated good discrimination for predicting 6-month mortality (AUROC 0.75‐0.81) and have been adapted for use in various settings, including nursing homes and community-based care [5,6,8]. Moreover, the Advanced Dementia Prognostic Tool score, specifically designed for residents with advanced dementia, reported an AUROC of 0.67 (95% CI 0.62‐0.72) [7,9]. More recent approaches include the Hospice Eligibility Prediction Index using hospital diagnoses and age (AUROC 0.838) [11] and Jorissen’s comprehensive model using 51 variables (AUROC 0.82), such as individual-level factors, LTCF-level factors, medication history, and health (hospital and medical practitioner) service use [12]. In the 2022 study by Chandra et al [14], an AUROC of 0.84 was reported for predicting mortality among hospitalized patients discharged to LTCFs, albeit this model also required extensive hospitalization data (eg, laboratory results and imaging findings). Importantly, models that depend on detailed hospital-derived diagnoses, medication histories, or longitudinal health service use may not consistently have at their disposal the data (or these data may not be readily linkable) they require to provide their predictions, potentially constraining their scalability and routine implementation in many care settings.

Standing apart from several preceding models, our model achieved strong discrimination using a smaller set of predictors, all of which are routinely documented in standard LTCF admission or early-stay assessments. This parsimony is a deliberate design choice that prioritizes real-world actionability: by avoiding reliance on hospital-derived diagnoses, medication histories, or granular use data, the model can be executed at, or shortly after, LTCF entry, a point at which timely prognostic awareness may highly meaningfully support ACP and goal-concordant care. This positioning is conceptually aligned with the established Changes in Health, End-Stage Disease, and Signs and Symptoms scale, which uses MDS- or International Resident Assessment Instrument–derived clinical indicators to predict short-term mortality and offers substantial improvement in prognostic precision [38]. Still, while the Changes in Health, End-Stage Disease, and Signs and Symptoms 3.0 [39] scale exhibits moderate discrimination for short-term death (AUROC of 0.72‐0.76 for 30- to 60-day periods), our model achieved a strong AUROC of 0.90 for 6-month mortality in the external validation. What this entails is that by leveraging machine learning to analyze functional trajectories and acute care history (ie, rather than relying primarily on symptomatic clusters), we can provide a resilient and accurate predictive signal supportive of longitudinal care coordination and resource allocation.

Our model was developed and temporally validated in Taiwanese LTCF residents, a population embedded within a long-term care system that includes home-based, community-based, and residential services. Moreover, the baseline characteristics and event rates of our cohort reflect a high-risk institutional population, characterized by substantial functional dependency and 6-month mortality rates of 22.1% in the development cohort and 28.7% in the temporal validation cohort. These mortality rates are broadly consistent with those reported in high-acuity nursing home and long-term care populations internationally [1,2]. Notwithstanding, direct comparisons should be interpreted cautiously, given cross-national differences in admission thresholds, assessment instruments, financing mechanisms, and availability of home- and community-based alternatives.

Most existing mortality prediction tools for LTCF or nursing home residents have been derived from Western cohorts, particularly US MDS-based populations [5-8,11,14]. However, differences in disease prevalence, health care use patterns, long-term care policy, family caregiving availability, cultural attitudes toward end-of-life care, and population health characteristics may limit the generalizability of Western-derived models to Asian LTCF settings [16]. By developing and temporally validating a prediction model using Taiwanese long-term care data, this study contributes region-specific evidence. It outlines that interpretable machine learning can achieve strong discrimination using locally relevant, routinely collected variables. It remains that the greater applicability of the model extends to LTCFs serving functionally dependent and medically complex older adults. External validation and, if necessary, local recalibration would be needed before applying the model to lower-acuity residential care, assisted living settings, or health systems with substantially different care delivery models.

Reliance on machine learning models (vs traditional logistic regression) reflects a deliberate trade-off between interpretability and predictive power. Although logistic regression offers a straightforward coefficient interpretation, it assumes linear relationships and cannot capture complex interactions among predictors without an explicit specification [40]. To address the interpretability challenge inherent in ensemble methods, we used SHAP analysis, which decomposes individual predictions into additive feature contributions grounded in cooperative game theory [35,36]. SHAP analysis revealed that frequent hospitalizations within 6 months, ADL impairment, and weight loss were the most influential predictors; these findings strongly align with established geriatric prognostic frameworks [3,41]. Functional decline (as measured by ADL scores) is a consistent and robust mortality predictor across diverse care settings [3,41]. Weight loss, particularly unintentional loss exceeding 5% of body weight, serves as a clinical marker of frailty, malnutrition, and underlying systemic illnesses [42]. The concordance between the SHAP-identified features and clinically validated risk factors enhances the model’s credibility, which may facilitate acceptance among care teams that may otherwise be skeptical of algorithm-driven recommendations.

Additionally, our model was developed and validated exclusively in Taiwanese LTCF residents, specifically and purposefully representing an Asian population. Meanwhile, most existing mortality prediction tools have been derived from Western cohorts, primarily using US MDS registries [5-8,11,14]. The ethnic and geographic differences in disease prevalence, health care use patterns, cultural attitudes toward end-of-life care, and nutritional status may limit the generalizability of Western-derived models to Asian contexts [16]. By developing a prediction model grounded in Taiwanese long-term care data, this study contributes region-specific evidence, emphasizing that interpretable machine learning approaches can achieve strong performance using locally relevant variables.

### Subgroup Performance and Algorithmic Fairness

A particularly noteworthy finding was the exceptional performance of the model (accuracy=0.85) among residents who demonstrated ADL improvement after LTCF admission. Functional improvement is typically clinically interpreted as a positive prognostic indicator, being used as a quality metric in institutional care [43,44]. However, a meaningful subset of residents with improving functional status may still face an elevated short-term mortality risk according to our findings, a pattern that may be difficult to identify using conventional clinical assessments alone.

Several mechanisms can explain this paradoxical association. First, some residents may experience transient functional recovery or “terminal lucidity,” referring to brief improvements in cognitive or physical function shortly before death, a phenomenon documented in the palliative care literature [45,46]. Second, residents with very low baseline ADL may show relative improvement from institutional rehabilitation while still harboring severe underlying comorbidities or physiological frailty, which may not be fully captured by functional measures alone [47,48]. Similarly, selection effects may operate such that residents admitted to LTCFs in acute decompensation states show functional “improvement” simply by returning toward their prior baseline, yet remain at a high mortality risk due to the severity of the precipitating illness or frailty [47,49].

From an algorithmic fairness perspective, this subgroup represents cases where human clinical judgment is most likely to underestimate risk, referring to situations sometimes termed “false reassurance” scenarios. The ability of the model to identify elevated mortality risk even in the presence of functional improvement has significant implications for ACP. This holds potential to enable timely goals-of-care conversations and referrals to palliative services for residents whose improving ADL trajectories could otherwise delay such discussions. The potential complementary role of algorithmic decision support is also emphasized here, showing that it does not replace clinical expertise but brings to the surface risk patterns that may be counterintuitive or masked by seemingly positive clinical indicators.

Subgroup analyses demonstrated the model’s stable performance across sex and age strata with minimal variation in discrimination (AUROC range 0.89‐0.90). Therefore, the model does not seem to systematically disadvantage specific demographic groups. This is key if we consider the ongoing concerns regarding algorithmic bias in health care AI [50]. Overall, the evidence from subgroups underlines the model’s robustness across key demographic strata, as well as the necessity of a prospective evaluation of whether risk signals in seemingly improving residents translate into better-timed, goal-concordant care when embedded in routine LTCF workflows.

### Potential Clinical and Operational Applications

Short-term mortality prediction in LTCFs may support several clinical and operational purposes, including ACP, palliative care assessment, medication review, family meetings, and resource planning [4]. Prognostic information can facilitate serious illness communication and goals-of-care discussions when embedded within structured clinical workflows, although evidence specific to LTCF settings remains lacking [13,51]. In addition, prior work in nursing home residents with advanced dementia has shown that prognostic tools may inform hospice eligibility discussions and care planning, while also underscoring the limitations of relying on prognostic models alone [7]. We put forward that the present model should be interpreted as a risk-stratification tool with potential clinical utility, rather than as evidence that model-guided care will necessarily improve outcomes [19].

Our model has several features that may support future implementation. First, it relies on variables available through LTCF assessment workflows, enabling risk stratification shortly after LTCF entry without requiring hospital record linkage or specialized testing. Second, it generates calibrated probability estimates, not categorical risk labels, allowing facilities to define thresholds according to local resources, resident and family preferences, and palliative care capacity. The threshold and decision-curve analyses also indicate that no single cutoff is optimal for all clinical purposes: lower thresholds may reduce missed high-risk residents but increase the number of residents flagged for review, whereas higher thresholds may reduce alert burden but miss more residents who die within 6 months. Thus, threshold selection should be linked to the intended clinical action and the relative consequences of false-negative versus false-positive classifications [52].

Third, SHAP-based explanations provide interpretable feature contributions that may help clinicians contextualize risk estimates using clinically meaningful domains, such as functional dependency, recent acute-care use, and nutritional decline. Some model features, such as weight loss and frequent hospitalization, should be interpreted as clinically recognizable signals that prompt further assessment, not that provide direction for treatment targets. For example, these signals may lead interdisciplinary teams to review nutritional status, symptom burden, recent care transitions, medication burden, or potentially reversible contributors to functional decline [53,54].

However, clinical utility cannot be assumed from discrimination and calibration alone. These metrics indicate that the model can rank residents by risk and estimate probabilities, while failing to demonstrate that using the model changes clinician behavior, improves care quality, or enhances resident- and family-centered outcomes [17,52]. This brings forth the need for future prospective impact and implementation studies before routine deployment [55]. Such studies should prespecify intended use, risk thresholds, and linked clinical actions; assess workflow integration and implementation outcomes such as acceptability, adoption, appropriateness, feasibility, fidelity, and sustainability; evaluate potential benefits and harms, including ACP uptake, palliative care referral, hospitalization, quality of end-of-life care, family experience, equity, and unintended consequences.

### Limitations

This study had several limitations. First, as this was a retrospective analysis of the JUBO database, the findings may be affected by documentation heterogeneity, documentation bias, and unmeasured facility-level effects. Differences in staffing patterns, care protocols, and documentation practices may influence both predictor recording and mortality risk. Facility-level clustering was also not explicitly modeled, and for selected binary or event-based variables, missing values were coded as absence according to the structure of routine LTCF documentation. This later-mentioned approach may conflate true clinical absence with incomplete recording. In sensitivity analyses, missingness in key clinical assessments was significantly associated with 6-month mortality, and facility-level documentation completeness showed significant heterogeneity across facilities and regions (Multimedia Appendices 14 and 15), raising the possibility of informative missingness, not just random documentation gaps. We thus need standardized assessment workflows, routine monitoring of facility-level missingness, and future evaluation of alternative missing-data strategies before prospective implementation of the model.

Second, data availability and temporal measurement limitations may have affected model granularity, benchmark selection, and predictor timing. Although we prioritized routinely available LTCF data to enhance real-world applicability, several potentially high-impact predictors, such as variables related to frailty or specific comorbidities, were excluded because missingness exceeded 30%, which may have reduced the model’s clinical granularity. In addition, direct comparison with established MDS- or International Resident Assessment Instrument–based clinical risk scores was not feasible because the required scoring components are not routinely used in Taiwanese LTCF workflows and were not fully available in the JUBO database. Some predictors also reflected information obtained during routine LTCF assessments after admission rather than strictly at the moment of admission. Although these variables were selected to reflect real-world longitudinal risk monitoring, their use may introduce temporal ambiguity or possible information leakage if the timing of risk estimation is not clearly aligned with predictor availability. Prospective implementation should ensure that each predictor is available before the risk estimate is generated.

Third, post-LTCF discharge vital status data were not consistently available in the JUBO database. Residents discharged or lost to follow-up before completion of the 6-month outcome window without subsequent status information were excluded from the primary analysis. Although the baseline characteristics of included and excluded residents were compared to assess potential selection bias, residual bias cannot be fully excluded because postdischarge mortality could not be directly observed.

Fourth, this study primarily modeled death within 6 months as a binary outcome. Despite adding cumulative or dynamic AUC analyses to evaluate discrimination across earlier time horizons, this approach provides only a complementary time-to-event discrimination assessment, hence not replacing formal survival modeling or fully accounting for censoring and time-to-death. Future studies should compare Cox regression, random survival forests, or gradient-boosted survival models with binary classification approaches to support dynamic risk prediction and updating during LTCF stay.

Fifth, the model’s transportability may be affected by cross-national differences in long-term care systems and temporal changes in baseline risk. LTCF admission thresholds vary across countries and may be shaped by financing mechanisms, family caregiving availability, home- and community-based service capacity, and national long-term care policies. In temporal external validation, quantitative calibration analyses showed modest underestimation of observed mortality, particularly at higher predicted-risk levels. This shows that calibration may shift over time or across care settings. External validation, calibration monitoring, and, if necessary, local or temporal recalibration are required before implementation in substantially different care settings.

Finally, the study period encompassed the COVID-19 pandemic, which may have affected LTCF mortality, admission case mix, hospitalization patterns, staffing, infection-control practices, and care delivery. Interpretations of the observed 6-month mortality rates of 22.1% in the development cohort and 28.7% in the temporal validation cohort should thus account for the aforementioned factors. Nevertheless, the model maintained comparable discriminative performance across the development and 2024 temporal validation cohorts, suggesting that the main person-level predictors retained prognostic value despite temporal changes in baseline risk. Validation in future postpandemic cohorts remains warranted.

### Future Directions

Future methodological work should go beyond periodic model updating and monitoring of calibration drift, also examining dynamic longitudinal prediction approaches. More recent temporal event prediction methods, including continuous estimation of temporal pattern completion for event prediction, may provide useful frameworks for incorporating repeated ADL assessments, weight trajectories, hospitalization events, falls, resident-reported outcomes, symptom burden, and evolving care needs into updated mortality risk estimates during LTCF stay.

### Conclusions

This study developed and temporally externally validated an interpretable machine learning model for predicting 6-month mortality among older LTCF residents in Taiwan using routinely collected LTCF assessment data. The model achieved strong discrimination and acceptable calibration without requiring hospital-based electronic health record linkage, suggesting potential feasibility for risk stratification within LTCF assessment workflows—albeit future prospective impact and implementation studies are recommended before actual deployment in real-world settings. Key predictors, including recent hospitalization, functional dependency and instability, and nutritional decline, were clinically coherent and aligned with geriatric prognostic domains. Prospective implementation studies, formal survival modeling, and external validation in other long-term care systems are needed to determine clinical impact, support dynamic risk updating, and assess broader transportability.

## Supplementary material

10.2196/94567 Multimedia Appendix 1 Literature review of predictors of mortality in long-term care settings.

10.2196/94567 Multimedia Appendix 2 Candidate predictors.

10.2196/94567 Multimedia Appendix 3 Assessment of data missingness across study cohorts.

10.2196/94567 Multimedia Appendix 4 Model candidates and qualitative comparison with key hyperparameters.

10.2196/94567 Multimedia Appendix 5 Definitions of evaluation metrics.

10.2196/94567 Multimedia Appendix 6 Characteristics of development cohort and excluded participants.

10.2196/94567 Multimedia Appendix 7 Quantitative calibration metrics of the predictive models in internal cross-validation and temporal external validation cohorts.

10.2196/94567 Multimedia Appendix 8 Risk-decile calibration tables of the hybrid of extreme gradient boosting and random forest model across validation cohorts.

10.2196/94567 Multimedia Appendix 9 Comparison of quantitative calibration metrics across all evaluated models.

10.2196/94567 Multimedia Appendix 10 Paired bootstrap test for hybrid of extreme gradient boosting and random forest and extreme gradient boosting.

10.2196/94567 Multimedia Appendix 11 Decision curve analysis.

10.2196/94567 Multimedia Appendix 12 Evaluation of alternative decision thresholds for the mortality prediction model.

10.2196/94567 Multimedia Appendix 13 Cumulative and dynamic area under the curve for time-to-event analysis.

10.2196/94567 Multimedia Appendix 14 Association between missing key clinical assessments and 6-month mortality (informative missingness analysis).

10.2196/94567 Multimedia Appendix 15 Overall documentation missingness and clinical outcomes stratified by facility size and geographic region.

10.2196/94567 Multimedia Appendix 16 Analysis source code and analytic scripts for model reproducibility.

10.2196/94567 Checklist 1 TRIPOD-AI checklist.

## Abbreviations

ACP: advance care planning
ADL: activities of daily living
AUC: area under the curve
AUROC: area under the receiver operating characteristic curve
HybridXGBRF: hybrid of extreme gradient boosting and random forest
LTCF: long-term care facility
MDS: minimum dataset
SHAP: Shapley Additive Explanations
TRIPOD-AI: Transparent Reporting of a Multivariable Prediction Model for Individual Prognosis Or Diagnosis-Artificial Intelligence
XGB: extreme gradient boosting
