# Supplementary material for: Development and Temporal External Validation of a Parsimonious, Interpretable Machine Learning Model for Predicting 6-Month Mortality in Long-Term Care Facilities: A Retrospective Cohort Study
Source: JMIR Aging. 2026 Sep 16;9:e94567. doi: 10.2196/94567 (PMC13581260; doi:10.2196/94567)
Supplement: Supplementary file 1 [file aging-v9-e94567-s001.docx]

Table S1. Literature review metrix of predictors for mortality in long-term care settings

| Study | Location | Study Design | Total cases / Average age | Men, % | Prognostic tool/model | Variables | Target Outcome | Discriminative ability |
| --- | --- | --- | --- | --- | --- | --- | --- | --- |
| Alrawi et al. (2013) | UK (East) | Prospective audit/Cohort study | N = 314 / Mean 84.2 years | 32% | Generalized Estimating Equations (GEE) (R 2.14.0) | MEWS (Modified Early Warning Score), eGFR, CRP, Polypharmacy, Age, Sex, Pneumonia, Fall history | In-patient mortality at 7 days | Odds Ratios only (no ROC AUC, F1, etc.)  - MEWS 6+ → OR 21.49  - eGFR <30 → OR 5.12  - CRP >100 → OR 2.47  - Polypharmacy ≥8 drugs → OR 0.30 |
| Levy et al. (2015) | USA | Retrospective cohort study | N = 140,699 / Not directly provided (Veterans) | Training 96.01%, Validation 96.03% | training (112,897 cases) and validation (27,832 cases) sets；naı¨ve Bayes approach | Hospital diagnoses (ICD-9 codes), Age on admission, Number of diagnoses (HELP Index) | 6-month mortality among nursing home residents | AUC=0.838 |
| Garcia-Gollarte et al. (2020) | Spain | Prospective observational cohort study | N = 531 / Mean 86.7 years | 24.70% | Cox proportional hazards model | Age, Sex, Functional dependence (Barthel), Cognitive impairment (Pfeiffer), Nutritional status (MNA), Comorbidities (Charlson, HBP, AF, pneumonia) | 15-month mortality | Harrell's C-index=0.689 |
| Chandra et al. (2022) | USA | Retrospective cohort study | N=11647 / Mean 79.2 years；Alive(n=9,803)/78.5 years, Death(n=1,844)/82.9 years | Alive 37.2%, Death 46.4% | Gradient Boosting Machine (GBM)；10-fold cross-validation | Age, sex, marital status, CCI；Number of hospital stays and emergency department (ED) visits in prior 6 months, ICU admission, Length of stay, Braden scale (pressure sore risk), Hendrich fall risk, Confusion Assessment Method (delirium)；hemoglobin, creatinine, sodium, potassium；Mobility status from PT records (e.g., sit-to-stand, ambulation) | 6-month all-cause death after discharge to a SNF(skilled nursing facility) | AUC (ROC): 0.81 (95% CI: 0.80–0.82) Sensitivity at 20% FPR: 62% (95% CI: 59–65%) Sensitivity at 30% FPR: 76% (95% CI: 74–79%) |
| Susnjak & Griffin (2023) | New Zealand & Australia | Retrospective machine learning model development | N = 11,944 / Mean 85.7 years | 38% | Cox Proportional Hazards (CoxPH), Elastic Net (EN), Ridge Regression (RR), Lasso, Gradient Boosting (GB), XGBoost (XGB) and Random Forest (RF) were tested in 20 experiments using different train/test splits at a 90/10 ratio. | Age category, gender, and predictors related to falls, health status, co-morbidities, cognitive function, mood state, nutritional status, mobility, smoking history, sleep, skin integrity, and continence. | survival at 6 months post-admission (older people admitted to residential age care) | AUC (ROC): 0.746 (95% CI 0.744-0.749) |
| Porock et al. (2010) | USA | Secondary analysis / Retrospective cohort study | N = 43,510 / Not explicitly stated | Not explicitly stated | Regression analysis | admission to the care home within three months; lost weight unintentionally in past three months; renal failure; chronic heart failure; poor appetite; male; dehydrated; short of breath; active cancer diagnosis; age; deteriorated cognitive skills in past three months; activities of daily living score.(Revised MDS Mortality Risk Index, MMRI-R) | 6-month mortality | ROC=0.76 |
| Zhang et al. (2023) | Global | Systematic review and meta‐analysis | 21 studies / Not applicable | Not applicable | Various prognostic models and indices | Study identified 28 indices that predicted the risk of all‐cause mortality from 14 days to 39 months among older adults in nursing homes. The most used predictors were age, sex, body weight, swallowing problem, congestive heart failure, shortness of breath, body mass index, and activities of daily living. | all-cause mortality (14 days to 39 months) | Of the 28 indices, 8 (29%) and 3 (11%) were internally and externally validated, respectively. None of the indices was validated in more than one cohort. Meta-analyzed C-statistic: 0.733 (CI : 0.669−0.797) |
| Thomas et al. (2019) | USA | Retrospective cohort study | N = 2,587,779 (Training 1,426,815; Testing 1,160,964) / Not explicitly stated | Training 36.88%, Testing 31.10% | Regression analysis | MDS 3.0 Mortality Risk Score (MRS3): 1) Cognitive and Physical Function, 2) Diagnoses, and 3) Health Conditions=>17 items: Severe Physical Impairment、Acute change in mental status、Shortness of breath、Dehydrated、Severe Cognitive Impairmenta、Internal bleeding、Swallowing disorder、 Vomiting、Fever、Unplanned weight loss、Heart Failure、Worst pressure ulcer(Slough or Necrotic)、Pneumonia、Any falls since admission or prior assessment、Viral Hepatitis、Hyponatremia、At least one stage or unstageable pressure ulcer) | 30-day and 60-day mortality | Results from logistic regression models suggest that the MRS3 performed well in predicting death within 30 and 60 days (C-Statistics of 0.744 (CI = 0.741-0.747) and 0.709 (CI = 0.706-0.711), respectively). |
| Vossius et al (2018) | Norway | Observational longitudinal study | N = 690 / Mean 84.4 years | 36.10% | Cox regression model | Demographic data (sex, age, and living status before admission to NH)、Diagnosis of dementia、CDR、NPI-NH、Physical Self-Maintenance Scale (PSMS)、General Medical Health Rating (GMHR) 、Charlson’s comorbidity index、BMI | 3-year mortality | Presented as Hazard Ratios (HR), AUC not reported. Mortality was associated with higher age and comorbidity at BL, and more severe dementia, higher ADL-dependency, less severe psychotic symptoms, and a lower BMI throughout the study period. |
| Muralitharan et al. (2021) | Global | Systematic scoping review | 24 studies included / Not applicable | Not applicable | Machine Learning-based Early Warning Systems (ML-based EWS) | Vital signs (Heart rate, respiratory rate, blood pressure, SpO2, temperature, consciousness) | Clinical deterioration, mortality, cardiac arrest, sepsis | AUC ranges: 0.57–0.97. Reported results suggest that machine learning–based early warning systems can achieve greater accuracy than aggregate-weighted early warning systems but several areas for further research were identified. |
| Chen et al. (2023) | USA | Retrospective cohort study | N = 1,489 (Dev 903, Val 586) / Mean 80.3 years (Dev), 83.78 years (Val) | Dev 27.35%, Val 30.89% | Logistic modeling | Age, sex, communication capacity, physical mobility, congestive heart failure, peripheral vascular disease, cancer, cerebrovascular disease, chronic renal disease and liver disease. | 1-year mortality | AUC: Dev 0.74, Val 0.68. For the validation cohort, the sensitivity and specificity were 0.79 and 0.53, respectively. |
| Veronese et al. (2024) | Global | Systematic review and meta-analysis | N = 2,398,856 (159 studies) / Mean 78 years | 49% | Multidimensional Prognostic Index (MPI) and other tools | Various clinical prognostic factors | Mortality, hospitalization, nursing home admission | Community-based populations: Tools with better long-term mortality prediction performance: (1) Health Assessment Tool: AUC = 0.87 (3-year follow-up); (2) Multidimensional Prognostic Index, MPI: C-index = 0.82 (15-year follow-up); (3) Other short- or medium-term (1–12 months) prediction accuracy is generally low (AUC < 0.70). Nursing homes: (1) MPI: 1-year mortality prediction AUC = 0.80 (good accuracy); (2) BMI + mini-mental State Examination + ADL combination: 5-year mortality prediction C-index = 0.78 (good accuracy) |
| Jorissen et al. (2024) | Australia | Retrospective cohort study | N = 116,192 / Median 85 years | 38.20% | Cox regression prediction model | 51 predictors (ADL, complex health conditions, medications, age, sex, malnutrition, etc.) | 90-day mortality | Harrell’s C-index = 0.773 (CI= 0.765–0.781). External validation C-index = 0.768. |
| Demonceau et al. (2023) | Belgium | Prospective longitudinal study | N = 533 / Mean 83.42 years | 26.60% | Multivariate Cox regression model | Data included clinical assessments (i.e., body composition, nutritional status, physical performance, level of dependence and cognition, frailty phenotype) as well as demographic information, number of medications and medical history. | 8-year mortality | Presented as Hazard Ratios (HR), C-index not reported. Younger age (HR = 1.04 (1.03–1.06)), higher BMI (HR = 0.96 (0.94–0.98)), higher score on the MMSE (HR = 0.97 (0.94–0.99)) and higher score on the SPPB (HR = 0.93 (0.90–0.97)) were protective factors against mortality. |
| Soraci et al (2025) | Italy | Prospective cohort study | N = 600 / Mean 80.4 years | 30.20% | Cox proportional hazards models | Anticholinergic burden was assessed at baseline using both CALS and ACB scales. MMSE and ADL. | 3-year mortality | C-index: CALS 0.783, ACB 0.781. CALS ≥ 2 was independently associated with cognitive impairment (odds ratio 1.84, 95% confidence interval 1.02–3.34), whereas ACB ≥ 2 was not. |
| Miller & Weissert (2020) | USA | Synthesis of longitudinal studies | 167 multivariate analyses from 78 articles / Not applicable | Not explicitly stated overall | Synthesis of various predictive models | 22 consistent risk factors (non-ADL physical function, illness severity, prior hospital use, etc.) | Institutionalization, functional impairment, and mortality | 22 risk factors consistently predict two or more outcomes, including three that predict all four: worse performance on physical function measures not based on activities of daily living, greater illness severity, and prior hospital use. |
| van der Steen et al. (2007) | Netherlands & USA | Cohort study (Validation) | N = 557 (Dutch 288, US 269) / Not explicitly stated | 25.30% | Logistic modeling | 12 MDS indicators (ADL dependence, male, cancer, congestive heart failure, oxygen therapy, shortness of breath, etc.) | 6-month mortality | AUROC: 0.64–0.65 |

**Reference**

Alrawi, Y. A., Parker, R. A., Harvey, R. C., Sultanzadeh, S. J., Patel, J., Mallinson, R., ... & Myint, P. K. (2013). Predictors of early mortality among hospitalized nursing home residents. QJM: An International Journal of Medicine, 106(1), 51-57.

Levy, C., Alemi, F., Wojtusiak, J., Sutton, B., & Williams, A. (2015). Predictors of 6-Month Mortality Among Nursing Home Residents: Are Diagnoses More Predictive than Functional Disability?Journal of Pain and Symptom Management, 49(2), 341.

García-Gollarte, J. F., García-Andrade, M. M., Santaeugenia-González, S. J., Solá Hermida, J. C., Baixauli-Alacreu, S., & Santabalbina, F. J. T. (2020). Risk factors for mortality in nursing home residents: an observational study. Geriatrics, 5(4), 71.

Chandra A, Takahashi PY, McCoy RG, Thorsteinsdottir B, Hanson GJ, Chaudhry R, Rahman PA, Storlie CB, Murphree DH Jr. Risk Prediction Model for 6-Month Mortality for Patients Discharged to Skilled Nursing Facilities. J Am Med Dir Assoc. 2022 Aug;23(8):1403-1408. doi: 10.1016/j.jamda.2022.01.069. Epub 2022 Feb 25. PMID: 35227666; PMCID: PMC9378493.

Susnjak, T., & Griffin, E. (2025). Towards clinical prediction with transparency: An explainable AI approach to survival modelling in residential aged care. Computer Methods and Programs in Biomedicine, 263, 108653.

Porock, D., Parker-Oliver, D., Petroski, G.F. et al. The MDS Mortality Risk Index: The evolution of a method for predicting 6-month mortality in nursing home residents. BMC Res Notes 3, 200 (2010). https://doi.org/10.1186/1756-0500-3-200

Zhang S, Zhang K, Chen Y, Wu C. Prediction models of all-cause mortality among older adults in nursing home setting: A systematic review and meta-analysis. Health Sci Rep. 2023 Jun 1;6(6):e1309. doi: 10.1002/hsr2.1309. PMID: 37275670; PMCID: PMC10233853.

Thomas, K. S., Ogarek, J. A., Teno, J. M., Gozalo, P. L., & Mor, V. (2019). Development and validation of the nursing home minimum data set 3.0 mortality risk score (MRS3). The Journals of Gerontology: Series A, 74(2), 219-225.

Vossius, C., Selbæk, G., Šaltytė Benth, J., & Bergh, S. (2018). Mortality in nursing home residents: A longitudinal study over three years. PloS one, 13(9), e0203480.

Muralitharan, S., Nelson, W., Di, S., McGillion, M., Devereaux, P. J., Barr, N. G., & Petch, J. (2021). Machine learning–based early warning systems for clinical deterioration: systematic scoping review. Journal of medical Internet research, 23(2), e25187.

Chen, X., Caplan, D. J., Comnick, C. L., Hartshorn, J., Shuman, S. K., & Xie, X. J. (2023). Development and validation of a nursing home mortality index to identify nursing home residents nearing the end of life in dental clinics. Special Care in Dentistry, 43(2), 125-135.

Veronese, N., Fazzari, A., Armata, M., Parisi, A., Parrinello, A., Petralia, V., ... & Barbagallo, M. (2024). Clinical prognostic factors for older people: A systematic review and meta-analysis. Ageing research reviews, 98, 102345.

Jorissen, R. N., Wesselingh, S. L., Whitehead, C., Maddison, J., Forward, J., Bourke, A., ... & Inacio, M. C. (2024). Predictors of mortality shortly after entering a long-term care facility. Age and Ageing, 53(5), afae098.

Demonceau, C., Buckinx, F., Reginster, J. Y., & Bruyère, O. (2023). Assessment of risk factors associated with long-term mortality in nursing homes: result from the SENIOR cohort. Aging Clinical and Experimental Research, 35(12), 2997-3005.

Soraci, L., Paparazzo, E., Aceto, M. A., Bruno, F., Serra Cassano, T., Lagrotteria, D., ... & Montesanto, A. (2025). CALS and ACB Scales are Associated with Physical and Cognitive Impairment and Predict Mortality in Nursing Home Residents. Drugs-Real World Outcomes, 12(3), 437-446.

Miller, E. A., & Weissert, W. G. (2000). Predicting elderly people’s risk for nursing home placement, hospitalization, functional impairment, and mortality: a synthesis. Medical care research and review, 57(3), 259-297.

van der Steen, J. T., Mitchell, S. L., Frijters, D. H., Kruse, R. L., & Ribbe, M. W. (2007). Prediction of 6-month mortality in nursing home residents with advanced dementia: validity of a risk score. Journal of the American Medical Directors Association, 8(7), 464-468.
