# Supplementary material for: Development and Temporal External Validation of a Parsimonious, Interpretable Machine Learning Model for Predicting 6-Month Mortality in Long-Term Care Facilities: A Retrospective Cohort Study
Source: JMIR Aging. 2026 Sep 16;9:e94567. doi: 10.2196/94567 (PMC13581260; doi:10.2196/94567)
Supplement: Supplementary file 2 [file aging-v9-e94567-s002.docx]

Table S1. Candidate predictor domains considered and their inclusion or exclusion status

| **Category** | **Predictor Variables** | **Content Definition** | **Availability Category** | **Timing of Acquisition** | **Included in Final Model** |
| --- | --- | --- | --- | --- | --- |
| **Demographics** |  |  |  |  |  |
|  | Age | Age (in years) at admission. | **At admission** | Recorded at the time of intake. | **Yes** |
|  | Sex | Male/Female. | **At admission** | Recorded at the time of intake. | **Yes** |
| **New resident’s basic info** |  |  |  |  |  |
|  | DNR^c^ | Do not resuscitate (Yes/No). | **At admission** | Recorded at the time of intake. | **Yes** |
|  | Dentures, tube feeding, and respiratory aids | Binary variables (Yes/No). *Combined from admission and daily records to minimize missingness.* | **At admission**/**During follow-up** | Prioritized baseline admission records; supplemented by daily nursing notes if initially missing. | **Yes** |
| **LTC**^c^ **assessment** |  |  |  |  |  |
|  | ADL^c^ | 10-domain score (range: 0–100). | **Shortly after admission**/**During follow-up** | First routine assessment within 7 days after admission. Subsequent reassessments every 3-6 months. | **Yes** |
|  | GDS-15^c^ | 15-item scale (score range: 0–15). | **Shortly after admission** | First routine assessment. | **No** (Excluded)*^a^* |
|  | SOF^c^ | 3-item scale (score range: 0–3). | **Shortly after admission** | First routine assessment. | **No** (Excluded)*^a^* |
|  | CIRS-G^c^ | 14-domain score (range: 0–56). | **Shortly after admission** | First routine assessment. | **No** (Excluded)*^a^* |
|  | Number of medications | Number of items, count (≥ 0). | **At admission** | Recorded at the time of intake. | **No** (Excluded)*^a^* |
|  | MNA-SF^c^ | 6-item scale (score range: 0–14). | **Shortly after admission** | First routine assessment. | **No** (Excluded)*^a^* |
| **Medical and Daily records** |  |  |  |  |  |
|  | Number of hospitalizations | Number of acute-care hospitalizations recorded before the risk-estimation time point. | **During follow-up** | New hospitalizations are continually recorded as they occur. | **Yes** |
|  | BW^c^ | Weight (kg) | **Shortly after admission**/**During follow-up** | First routine assessment within 7 days after admission. Reassessments conducted routinely. | **Yes** |
|  | Fall count | Number of events, count (≥ 0). | **During follow-up** | Documented as needed during daily care. | **Yes** |
|  | GCS^c^ | 3 parameters; best eye response (E); best verbal response (V); best motor response (M); E, V, M; score range: 3–15. | **Shortly after admission**/**During follow-up** | First routine assessment within 7 days after admission. | **Yes** |
|  | Lab data | Data of eGFR & C-reactive Protein (CRP). | **During follow-up** | Intermittently recorded during medical visits. | **No** (Excluded)*^b^* |

^a^ Excluded from the final model. These candidate domains exhibited high data incompleteness rates (>50%), exceeding our prespecified exclusion threshold of >30% missingness.

^b^ Excluded from the final model. Laboratory data and certain vital signs were excluded because some institutions do not provide fully structured electronic data for these variables.

^c^ ADL = activities of daily living; BW = body weight; CIRS-G = modified cumulative illness rating scale for geriatric patients; DNR = do not resuscitate; GCS = Glasgow Coma Scale; GDS-15 = Geriatric Depression Scale 15 score; LTC = Long-term care; MNA-SF = Mini-Nutritional Assessment-Short-Form; SOF = Study of Osteoporotic Fracture score.
