# Supplementary material for: Development and Temporal External Validation of a Parsimonious, Interpretable Machine Learning Model for Predicting 6-Month Mortality in Long-Term Care Facilities: A Retrospective Cohort Study
Source: JMIR Aging. 2026 Sep 16;9:e94567. doi: 10.2196/94567 (PMC13581260; doi:10.2196/94567)
Supplement: Supplementary file 3 [file aging-v9-e94567-s003.docx]

Table S1. Assessment of data missingness across study cohorts

| **Variable name** | **Development cohort (N = 23,901)** | | **External validation cohort (N = 6216)** | | |
| --- | --- | --- | --- | --- | --- |
|  | Missing number | Percent | Missing Number | Percent |  |
| Age | 0 | 0% | 0 | 0% |  |
| Sex | 0 | 0% | 0 | 0% |  |
| CIRS-G^a^ | 18,619 | 77.9% | 3962 | 63.7% |  |
| DNR notes | 14,124 | 59.1% | 3384 | 54.4% |  |
| ADL^a^ | 0 | 0% | 0 | 0% |  |
| Second ADL | 5145 | 21.5% | 1562 | 25.1% |  |
| GCS^a^ (EVM^a^) | 2229 | 9.3% | 558 | 9.0% |  |
| Dentures | 2229 | 9.3% | 558 | 9.0% |  |
| Tube feeding | 2229 | 9.3% | 558 | 9.0% |  |
| Respiratory support | 2229 | 9.3% | 558 | 9.0% |  |
| History of falls | 2229 | 9.3% | 558 | 9.0% |  |
| BW^a^ | 3735 | 15.6% | 930 | 15.0% |  |
| Number of admissions in 6 months | 0 | 0% | 0 | 0% |  |
| Institutional regions | 0 | 0% | 0 | 0% |  |

^a^ ADL, activities of daily living; BW, body weight; CIRS-G, Modified Cumulative Illness Rating Scale for Geriatric Patients; DNR, do-not-resuscitate; EVM, best eye response (E), best verbal response (V), and best motor response (M); GCS, Glasgow Coma Scale.
