# Supplementary material for: Development and Temporal External Validation of a Parsimonious, Interpretable Machine Learning Model for Predicting 6-Month Mortality in Long-Term Care Facilities: A Retrospective Cohort Study
Source: JMIR Aging. 2026 Sep 16;9:e94567. doi: 10.2196/94567 (PMC13581260; doi:10.2196/94567)
Supplement: Supplementary file 4 [file aging-v9-e94567-s004.docx]

Table S1. Model candidates and qualitative comparisons with key hyperparameters

| **Model** | **Type** | **Qualitative comparison and key hyperparameter setting** |
| --- | --- | --- |
| HybridXGBRF (Our Approach) | Ensemble | This model blends an XGBoost classifier and a Random Forest Classifier at the probability level, computing a weighted probability: p = α·p_xgb + (1−α)·p_rf, where α ∈ [0,1] controls the contribution of XGBoost versus Random Forest. XGBoost captures nonlinear interactions and high-order feature interactions, while Random Forests add robustness to noisy or sparse signals. We tune α on validation and keep the selected α for external testing. |
| XGB Classifier | Gradient Boosting | This model uses gradient-boosted decision trees and learns nonlinear feature interactions, performing well when the signal depends on combinations of clinical status and trend features. In our run, we set n_estimators = 200, learning_rate = 0.01, max_depth = 5, eval_metric = logloss, and random_state = 42 (other parameters follow library defaults unless specified). |
| Random Forest Classifier | Bagging | This model uses bagged decision trees and reduces variance through averaging, often delivering stable performance across mixed feature types (binary event flags and continuous summaries). In our run, we set n_estimators = 100 and random_state = 42 (other parameters use default values unless specified). |
| Logistic Regression (max_iter=200) | Linear | This model provides a linear baseline and produces simple, interpretable decision boundaries, working well when risk increases approximately linearly with the features (e.g., age and ADL level). In our run, we set max_iter = 200 and keep the other settings at the sklearn defaults (e.g., L2 regularization with the default solver). |
| Logistic Regression (max_iter=1000) | Linear | This model matches the logistic regression baseline while allowing for more optimization steps to achieve convergence. In our run, we set max_iter = 1000 and keep other settings at sklearn defaults (e.g., L2 regularization with the default solver). |
| Ridge | Regularized Linear | This pipeline uses StandardScaler + LogisticRegression with L2 (ridge) penalty, shrinking coefficients and helping when predictors are collinear (familiar with first/last/max/min/diff features). In our run, we use penalty = l2, solver = saga, max_iter = 1000, and random_state = 42 (C uses the default value unless specified). |
| Lasso | Regularized Linear | This pipeline uses StandardScaler and LogisticRegression with an L1 (Lasso) penalty, encouraging sparsity and being capable of dropping weak predictors by pushing some coefficients to zero. In our run, we use penalty = “l1,” solver = “saga,” max_iter = 1000, and random_state = 42 (C uses the default value unless specified). |
| Elastic | Regularized Linear | This pipeline uses StandardScaler + LogisticRegression with an elastic-net penalty, which combines L1 and L2 behavior. It can keep correlated predictors while still performing selection. In our run, we use penalty = elasticnet, solver = saga, l1_ratio = 0.5, max_iter = 1000, and random_state = 42 (C uses the default value unless specified). |
