# Supplementary material for: Development and Temporal External Validation of a Parsimonious, Interpretable Machine Learning Model for Predicting 6-Month Mortality in Long-Term Care Facilities: A Retrospective Cohort Study
Source: JMIR Aging. 2026 Sep 16;9:e94567. doi: 10.2196/94567 (PMC13581260; doi:10.2196/94567)
Supplement: Supplementary file 5 [file aging-v9-e94567-s005.docx]

Table S1. Definitions of evaluation metrics

| **Evaluation index** | **Description/definition** |
| --- | --- |
| True positive | The number of patients who had an event and were predicted by the model to have one. |
| False positive | The number of patients who did not have an event but were predicted by the model to have one. |
| True negative | The number of patients who did not have an event and were also not predicted by the model to have one. |
| False negative | The number of patients who had an event but were not predicted by the model to have one. |
| Precision | The proportion of true positive results among all instances predicted as positive: TP / (TP + FP) |
| Recall | The proportion of true positive results among all actual positive instances: TP / (TP + FN) |
| Sensitivity | Identical to recall; the ability of a test to correctly identify those with the disease. |
| Specificity | The ability of a test to correctly identify those without the disease (i.e., true negative rate): TN / (TN + FP) |
| F1 Score | The harmonic mean between precision and recall, using the following formula: F1 = 2 / (1 / Precision + 1 / Recall) |
| Accuracy | The proportion of total correct predictions (both true positives and true negatives) among the total number of cases examined: (TP + TN) / (TP + TN + FP + FN) |
| AUROC | Area under the receiver operating characteristic curve, which represents the probability that a classifier will rank a randomly chosen positive instance higher than a randomly chosen negative one. |
| Brier score | A score that measures the accuracy of probabilistic predictions; it is the mean squared difference between predicted probabilities and actual outcomes. |
