# Supplementary material for: Development and Temporal External Validation of a Parsimonious, Interpretable Machine Learning Model for Predicting 6-Month Mortality in Long-Term Care Facilities: A Retrospective Cohort Study
Source: JMIR Aging. 2026 Sep 16;9:e94567. doi: 10.2196/94567 (PMC13581260; doi:10.2196/94567)
Supplement: Supplementary file 6 [file aging-v9-e94567-s006.docx]

Table S1. Characteristics of the development cohort and excluded subjects

| **Characteristic** | | | **Development cohort (N = 23,901)** | **%** | **Excluded subjects (Unable to ascertain the 6-month mortality outcome) (n = 19,756)** | **%** | **P** |
| --- | --- | --- | --- | --- | --- | --- | --- |
| Age, mean ± SD^a^ | | | 79.3±11.8 |  | 76.6±14.9 |  | **< .001** |
| **Age group** |  | |  |  |  |  | **< .001** |
|  | 65–69 | | 1926 | 8.1% | 1943 | 9.8% |  |
|  | 70–74 | | 2794 | 11.7% | 2636 | 13.3% |  |
|  | 75–79 | | 3112 | 13.0% | 2860 | 14.5% |  |
|  | 80–84 | | 4345 | 18.2% | 3605 | 18.2% |  |
|  | 85–89 | | 4734 | 19.8% | 3068 | 15.5% |  |
|  | 90+ | | 4448 | 18.6% | 2573 | 13.0% |  |
| **Sex** |  | |  |  |  |  | **.02** |
|  | Male | | 11,746 | 49.1% | 9929 | 50.3% |  |
|  | Female | | 12,155 | 50.9% | 9827 | 49.7% |  |
| CIRS-G^a^, mean ± SD | | | 1.74±0.59 |  | NA |  |  |
| DNR^a^ notes (+) | | | 9777 | 40.9% | 5032 | 25.5% | **< .001** |
| **ADL**^a^ |  | |  |  |  |  |  |
|  | First score, mean ± SD | | 28.6±31.7 |  | 32.6±30.9 |  | **< .001** |
|  | Max score in six months, mean ± SD | | 31.0±32.7 |  | 34.0±31.6 |  | **< .001** |
|  | Last score, mean ± SD | | 28.5±31.8 |  | 30.6±31.1 |  | **< .001** |
| GCS^a,b^ (EVM), mean ± SD | | | 7.7±5.3 |  | 7.1±5.3 |  | **< .001** |
| Dentures | | | 3918 | 16.4% | 2155 | 18.3% | **< .001** |
| Tube feeding | | | 2081 | 8.7% | 978 | 8.3% | .18 |
| Respiratory support | | | 848 | 3.5% | 407 | 3.4% | .63 |
| History of falls | | | 1197 | 5.0% | NA |  | NA |
| BW^a^, mean ± SD | | | 51.4±12.0 |  | 55.2±12.9 |  | **< .001** |
| **Institutional regions** | |  |  |  |  |  | **< .001** |
|  | | North | 2245 | 9.39% | 2112 | 10.69% |  |
|  | | Central | 4754 | 19.89% | 4019 | 20.34% |  |
|  | | South | 16,603 | 69.46% | 13,447 | 68.07% |  |
|  | | East | 299 | 1.25% | 174 | 0.88% |  |
| **Institution scale** | | |  |  |  |  | .93 |
|  | | Small (<50 beds) | 311 | 63.08% | 363 | 61.95% |  |
|  | | Medium (50-150 beds) | 148 | 30.02% | 182 | 31.06% |  |
|  | | Large (>150 beds) | 34 | 6.90% | 41 | 7.00% |  |

**Unable to ascertain 6-month mortality outcome:** residents discharged or lost to follow-up before completion of the 6-month outcome window whose post-discharge vital status was unavailable in the JUBO database. Residents who died within 6 months while under LTCF follow-up were included as outcome events.

^a^ ADL, activities of daily living; BW, body weight; CIRS-G, Modified Cumulative Illness Rating Scale for Geriatric Patients; DNR, do not resuscitate; GCS, Glasgow Coma Scale; SD, standard deviation.

^b^ GCS scores between 3 and 15; it comprises three parameters: best eye response (E), best verbal response (V), and best motor response (M).

Table S2. Characteristics of the development cohort and excluded subjects **(No available baseline ADL assessment)**

| **Characteristic** | | **Development cohort (N = 23,901)** | **%** | **Excluded subjects (No available baseline ADL assessment) (n = 11,449)** | **%** | **P** |
| --- | --- | --- | --- | --- | --- | --- |
| Age, mean ± SD^a^ | | 79.3±11.8 |  | 78.5±17.0 |  | **< 0.001** |
| **Age group** |  |  |  |  |  | **< 0.001** |
|  | 65–69 | 1926 | 8.1% | 868 | 7.6% |  |
|  | 70–74 | 2794 | 11.7% | 1234 | 10.8% |  |
|  | 75–79 | 3112 | 13.0% | 1435 | 12.5% |  |
|  | 80–84 | 4345 | 18.2% | 1939 | 16.9% |  |
|  | 85–89 | 4734 | 19.8% | 2116 | 18.5% |  |
|  | 90+ | 4448 | 18.6% | 2301 | 20.1% |  |
| **Sex** |  |  |  |  |  | 0.73 |
|  | Male | 11,746 | 49.1% | 5649 | 49.3% |  |
|  | Female | 12,155 | 50.9% | 5800 | 50.7% |  |
| CIRS-G^a^, mean ± SD | | 1.74±0.59 |  | 1.49±0.55 |  | **< 0.001** |
| DNR^a^ notes (+) | | 9777 | 40.9% | 3269 | 28.6% | **< 0.001** |
| GCS^a,b^ (EVM), mean ± SD | | 7.7±5.3 |  | 7.0±4.9 |  | **< 0.001** |
| Dentures | | 3918 | 16.4% | 44 | 0.4% | **< 0.001** |
| Tube feeding | | 2081 | 8.7% | 23 | 0.2% | **< 0.001** |
| Respiratory support | | 848 | 3.5% | 12 | 0.1% | **< 0.001** |
| History of falls | | 1197 | 5.0% | 5 | 0.04% | **< 0.001** |
| BW^a^, mean ± SD | | 51.4±12.0 |  | 52.1±11.3 |  | **< 0.001** |
| Number of admissions in six months, mean ± SD | | 0.5±1.0 |  | 0.13±0.48 |  | **< 0.001** |
| **Institutional regions** | |  |  |  |  | **< 0.001** |
|  | North | 2245 | 9.39% | 1347 | 11.77% |  |
|  | Central | 4754 | 19.89% | 3987 | 34.82% |  |
|  | South | 16,603 | 69.46% | 5853 | 51.12% |  |
|  | East | 299 | 1.25% | 230 | 2.01% |  |
| **Institution scale** |  |  |  |  |  | 0.90 |
|  | Small (<50 beds) | 311 | 63.08% | 332 | 61.82% |  |
|  | Medium (50-150 beds) | 148 | 30.02% | 165 | 30.73% |  |
|  | Large (>150 beds) | 34 | 6.90% | 40 | 7.45% |  |

**No available baseline ADL assessment:** residents with no recorded activities of daily living (ADL) assessment at admission and therefore no baseline ADL information available for feature construction. Residents with a recorded ADL assessment but partially missing ADL items were retained and handled using the prespecified imputation procedure.

^a^ ADL, activities of daily living; BW, body weight; CIRS-G, modified cumulative illness rating scale for geriatric patients; DNR, do not resuscitate; GCS, Glasgow Coma Scale; SD, standard deviation.

^b^ GCS scores between 3 and 15; it comprises three parameters: best eye response (E), best verbal response (V), and best motor response (M).
