# Supplementary material for: Development and Temporal External Validation of a Parsimonious, Interpretable Machine Learning Model for Predicting 6-Month Mortality in Long-Term Care Facilities: A Retrospective Cohort Study
Source: JMIR Aging. 2026 Sep 16;9:e94567. doi: 10.2196/94567 (PMC13581260; doi:10.2196/94567)
Supplement: Supplementary file 7 [file aging-v9-e94567-s007.docx]

Table S1. Quantitative calibration metrics of the predictive models in the internal cross-validation and temporal external validation cohorts

| **Calibration metric** | **Internal cross-validation (Estimate with 95% CI)** | **Temporal external validation (Estimate with 95% CI)** |
| --- | --- | --- |
| **Calibration intercept** | 0.197 (0.160–0.235) | 0.516 (0.447–0.597) |
| **Calibration slope** | 1.214 (1.182–1.249) | 1.271 (1.217–1.342) |
| **Observed/expected (O/E) ratio** | 1.000 (0.990–1.011) | 1.128 (1.108–1.149) |
| **Brier score** | 0.101 (0.099–0.103) | 0.112 (0.108–0.116) |
