# Supplementary material for: Development and Temporal External Validation of a Parsimonious, Interpretable Machine Learning Model for Predicting 6-Month Mortality in Long-Term Care Facilities: A Retrospective Cohort Study
Source: JMIR Aging. 2026 Sep 16;9:e94567. doi: 10.2196/94567 (PMC13581260; doi:10.2196/94567)
Supplement: Supplementary file 8 [file aging-v9-e94567-s008.docx]

Table S1. Internal Cross-Validation Cohort

| **Decile (Risk Stratum)** | **N (Residents)** | **Deaths (Observed)** | **Observed Risk** | **Mean Predicted Risk** | **Predicted Risk Range (Min - Max)** |
| --- | --- | --- | --- | --- | --- |
| 1 (Lowest Risk) | 2391 | 13 | 0.005 | 0.019 | (0.008-0.025) |
| 2 | 2390 | 50 | 0.021 | 0.031 | (0.025-0.038) |
| 3 | 2390 | 71 | 0.030 | 0.046 | (0.038-0.053) |
| 4 | 2390 | 108 | 0.045 | 0.061 | (0.053-0.071) |
| 5 | 2390 | 186 | 0.078 | 0.091 | (0.071-0.118) |
| 6 | 2393 | 313 | 0.131 | 0.154 | (0.118-0.185) |
| 7 | 2387 | 503 | 0.211 | 0.212 | (0.185-0.246) |
| 8 | 2390 | 692 | 0.290 | 0.298 | (0.246-0.369) |
| 9 | 2390 | 1270 | 0.531 | 0.499 | (0.369-0.639) |
| 10 (Highest Risk) | 2390 | 2066 | 0.864 | 0.795 | (0.640-0.930) |

Table S2. Temporal External Validation Cohort

| **Decile (Risk Stratum)** | **N (Residents)** | **Deaths (Observed)** | **Observed Risk** | **Mean Predicted Risk** | **Predicted Risk Range (Min - Max)** |
| --- | --- | --- | --- | --- | --- |
| 1 (Lowest Risk) | 623 | 7 | 0.011 | 0.020 | 0.010 – 0.027 |
| 2 | 621 | 9 | 0.014 | 0.034 | 0.027 – 0.041 |
| 3 | 622 | 24 | 0.039 | 0.050 | 0.041 – 0.057 |
| 4 | 621 | 46 | 0.074 | 0.069 | 0.057 – 0.085 |
| 5 | 621 | 77 | 0.124 | 0.117 | 0.085 – 0.156 |
| 6 | 622 | 123 | 0.198 | 0.186 | 0.156 – 0.213 |
| 7 | 621 | 188 | 0.303 | 0.248 | 0.213 – 0.286 |
| 8 | 623 | 258 | 0.414 | 0.367 | 0.286 – 0.478 |
| 9 | 620 | 461 | 0.744 | 0.608 | 0.479 – 0.740 |
| 10 (Highest Risk) | 622 | 588 | 0.945 | 0.840 | 0.740 – 0.927 |
