# Supplementary material for: Development and Temporal External Validation of a Parsimonious, Interpretable Machine Learning Model for Predicting 6-Month Mortality in Long-Term Care Facilities: A Retrospective Cohort Study
Source: JMIR Aging. 2026 Sep 16;9:e94567. doi: 10.2196/94567 (PMC13581260; doi:10.2196/94567)
Supplement: Supplementary file 9 [file aging-v9-e94567-s009.docx]

Table S1. Comparison of Quantitative Calibration Metrics Across All Evaluated Models

| **Cohort** | **Model** | **Calibration Intercept** | **Calibration Slope** | **O/E Ratio** | **Brier Score** |
| --- | --- | --- | --- | --- | --- |
| **Internal CV** | **HybridXGBRF (Our Approach)** | **0.197** | **1.214** | **1.000** | **0.101** |
| Internal CV | XGBClassifier | 0.380 | 1.393 | 1.001 | 0.103 |
| Internal CV | RandomForestClassifier | -0.241 | 0.785 | 0.974 | 0.106 |
| Internal CV | Logistic Regression | 0.029 | 1.029 | 1.000 | 0.116 |
| Internal CV | Ridge | 0.054 | 1.054 | 1.000 | 0.116 |
| Internal CV | Lasso | 0.039 | 1.039 | 1.000 | 0.116 |
| Internal CV | Elastic | 0.047 | 1.047 | 1.000 | 0.116 |
| **Temporal external validation** | **HybridXGBRF (Our Approach)** | **0.516** | **1.271** | **1.128** | **0.112** |
| Temporal external validation | XGBClassifier | 0.726 | 1.456 | 1.152 | 0.117 |
| Temporal external validation | RandomForestClassifier | 0.073 | 0.824 | 1.095 | 0.119 |
| Temporal external validation | Logistic Regression | 0.320 | 1.066 | 1.133 | 0.129 |
| Temporal external validation | Ridge | 0.341 | 1.088 | 1.136 | 0.130 |
| Temporal external validation | Lasso | 0.330 | 1.077 | 1.135 | 0.129 |
| Temporal external validation | Elastic | 0.336 | 1.083 | 1.135 | 0.129 |
