# Supplementary material for: Development and Temporal External Validation of a Parsimonious, Interpretable Machine Learning Model for Predicting 6-Month Mortality in Long-Term Care Facilities: A Retrospective Cohort Study
Source: JMIR Aging. 2026 Sep 16;9:e94567. doi: 10.2196/94567 (PMC13581260; doi:10.2196/94567)
Supplement: Supplementary file 10 [file aging-v9-e94567-s010.docx]

Table S1. Paired bootstrap test for HybridXGBRF and XGBoost

| **Evaluation Cohort** | **Model A (Our Approach)** | **Model B (Benchmark)** | **AUROC A** | **AUROC B** | **Paired AUROC Difference (A–B)** | **95% Confidence Interval of Difference** | **Bootstrap P-value** |
| --- | --- | --- | --- | --- | --- | --- | --- |
| **Development cohort** | HybridXGBRF | XGBClassifier | 0.888 | 0.885 | 0.003 | (0.002 – 0.005) | **<0.001** |
| **External Validation** | HybridXGBRF | XGBClassifier | 0.898 | 0.894 | 0.004 | (0.001 – 0.006) | **0.003** |
