# Supplementary material for: Development and Temporal External Validation of a Parsimonious, Interpretable Machine Learning Model for Predicting 6-Month Mortality in Long-Term Care Facilities: A Retrospective Cohort Study
Source: JMIR Aging. 2026 Sep 16;9:e94567. doi: 10.2196/94567 (PMC13581260; doi:10.2196/94567)
Supplement: Supplementary file 12 [file aging-v9-e94567-s012.docx]

Table S1. Evaluation of Alternative Decision Thresholds for the Mortality Prediction Model

| Threshold | Net Benefit | Accuracy | Sensitivity | Specificity | PPV | NPV | F1 | TP | FP | TN | FN |
| --- | --- | --- | --- | --- | --- | --- | --- | --- | --- | --- | --- |
| 0.1 | 0.234 | 0.678 | 0.934 | 0.576 | 0.469 | 0.956 | 0.624 | 1663 | 1882 | 2553 | 118 |
| 0.2 | 0.201 | 0.779 | 0.861 | 0.746 | 0.577 | 0.930 | 0.691 | 1533 | 1126 | 3309 | 248 |
| 0.3 | 0.172 | 0.838 | 0.723 | 0.884 | 0.714 | 0.888 | 0.719 | 1288 | 515 | 3920 | 493 |
| 0.4 | 0.153 | 0.852 | 0.641 | 0.936 | 0.801 | 0.867 | 0.712 | 1142 | 283 | 4152 | 639 |
| 0.5 | 0.135 | 0.848 | 0.567 | 0.961 | 0.855 | 0.847 | 0.682 | 1010 | 171 | 4264 | 771 |
| 0.6 | 0.114 | 0.835 | 0.482 | 0.977 | 0.895 | 0.825 | 0.627 | 859 | 101 | 4334 | 922 |
| 0.7 | 0.088 | 0.811 | 0.368 | 0.989 | 0.933 | 0.796 | 0.528 | 656 | 47 | 4388 | 1125 |
| 0.8 | 0.058 | 0.782 | 0.252 | 0.995 | 0.953 | 0.768 | 0.399 | 449 | 22 | 4413 | 1332 |
| 0.9 | 0.010 | 0.724 | 0.036 | 1.000 | 1.000 | 0.721 | 0.069 | 64 | 0 | 4435 | 1717 |
