# Supplementary material for: Development and Temporal External Validation of a Parsimonious, Interpretable Machine Learning Model for Predicting 6-Month Mortality in Long-Term Care Facilities: A Retrospective Cohort Study
Source: JMIR Aging. 2026 Sep 16;9:e94567. doi: 10.2196/94567 (PMC13581260; doi:10.2196/94567)
Supplement: Supplementary file 13 [file aging-v9-e94567-s013.docx]

Table S1. Cumulative and Dynamic Area Under the Curve (AUC) for Time-to-Event Analysis

| Development cohort | | | |
| --- | --- | --- | --- |
| Month | Cases by time t | Controls event-free beyond t | Cumulative/Dynamic AUC |
| 1 | 1811 | 22090 | 0.922 |
| 2 | 2928 | 20973 | 0.923 |
| 3 | 3707 | 20194 | 0.917 |
| 4 | 4334 | 19567 | 0.906 |
| 5 | 4848 | 19053 | 0.898 |
| External validation cohort | | | |
| 1 | 729 | 5487 | 0.916 |
| 2 | 1136 | 5080 | 0.924 |
| 3 | 1372 | 4844 | 0.918 |
| 4 | 1552 | 4664 | 0.908 |
| 5 | 1682 | 4534 | 0.902 |
