# Supplementary material for: Development and Temporal External Validation of a Parsimonious, Interpretable Machine Learning Model for Predicting 6-Month Mortality in Long-Term Care Facilities: A Retrospective Cohort Study
Source: JMIR Aging. 2026 Sep 16;9:e94567. doi: 10.2196/94567 (PMC13581260; doi:10.2196/94567)
Supplement: Supplementary file 14 [file aging-v9-e94567-s014.docx]

Table S1. Association Between Missing Key Clinical Assessments and 6-Month Mortality (Informative Missingness Analysis)

| **Cohort** | **Missingness Indicator** | **Missing Rate, % (n/Total N)** | **Adjusted Odds Ratio (OR) for Mortality** | ***P*** |
| --- | --- | --- | --- | --- |
| **Development Cohort** (N = 23,901) |  |  |  |  |
|  | Missing subsequent ADL score | 21.5% (5,145) | **13.7** | **<0.001** |
|  | Missing initial body weight | 15.6% (3,735) | **3.0** | **<0.001** |
|  | Missing GCS score | 9.3% (2,229) | **1.2** | **<0.001** |
| **External Validation Cohort** (N = 6,216) |  |  |  |  |
|  | Missing subsequent ADL score | 25.1% (1,562) | **16.6** | **<0.001** |
|  | Missing initial body weight | 15.0% (930) | **3.1** | **<0.001** |
|  | Missing GCS score | 9.0% (558) | **1.3** | **0.01** |
