# Supplementary material for: Development and Temporal External Validation of a Parsimonious, Interpretable Machine Learning Model for Predicting 6-Month Mortality in Long-Term Care Facilities: A Retrospective Cohort Study
Source: JMIR Aging. 2026 Sep 16;9:e94567. doi: 10.2196/94567 (PMC13581260; doi:10.2196/94567)
Supplement: Supplementary file 15 [file aging-v9-e94567-s015.docx]

Table S1. Overall Documentation Missingness and Clinical Outcomes Stratified by Facility Size and Geographic Region

| **Variable** | **N facilities** | **N residents** | **Overall missing percent** | **6-Month Mortality Rate** |
| --- | --- | --- | --- | --- |
| **Facility Size** |  |  |  |  |
| Small (<50 beds) | 311 | 12161 | 7.7% | 20.9% |
| Medium (50-150 beds) | 148 | 8412 | 7.9% | 24.8% |
| Large (>150 beds) | 34 | 3328 | 8.0% | 19.4% |
| **Facility Region** |  |  |  |  |
| North | 54 | 2245 | 12.1% | 16.8% |
| Central | 100 | 4754 | 8.8% | 23.6% |
| South | 332 | 16603 | 7.0% | 22.5% |
| East | 7 | 299 | 5.5% | 10.0% |

Overall Missing Rate (%): This metric represents the average proportion of missing data cells across all predictive features within the specified stratum, rather than the percentage of residents with any missing data.

**A global chi-square test of independence across all 493 individual facilities confirmed highly significant inter-institutional heterogeneity in overall documentation completeness (χ2 =140785.98, P<.001, Cramer's V = 0.451).**
