# Supplementary material for: Development and Temporal External Validation of a Parsimonious, Interpretable Machine Learning Model for Predicting 6-Month Mortality in Long-Term Care Facilities: A Retrospective Cohort Study
Source: JMIR Aging. 2026 Sep 16;9:e94567. doi: 10.2196/94567 (PMC13581260; doi:10.2196/94567)
Supplement: Supplementary file 16 [file aging-v9-e94567-s016.docx]

Source Code and Analytic Scripts for Model Reproducibility

1. **Data Availability and Code Repository**

The analytic scripts, preprocessing procedures, model specifications, and generated aggregate outputs are publicly available to support transparency and reproducibility. Individual-level resident data are not included in the repository because of privacy and data-use restrictions. The repository contains code, configuration files, documentation, and nonidentifiable aggregate outputs only. **Repository URL:** <https://github.com/peculab/AI4JUBO>

2. **System Requirements and Dependencies**

- **Environment:** Analyses were developed and executed in Python, including use within the Google Colaboratory cloud computing environment.
- **Programming Language:** Python
- **Key Packages:** xgboost, scikit-learn, shap, pandas, numpy, plotly, openpyxl, and kaleido.
- **Reproducibility**: A fixed random seed (random_state = 42) was used for stochastic components, including stratified cross-validation splitting, XGBoost and random forest model construction, bootstrap resampling, and SHAP sampling procedures.

3. **Code Structure and Analytical Workflow**

The repository includes a primary Jupyter Notebook and modular Python scripts that implement the analytic workflow. The workflow includes the following components:

- Data Preprocessing: Scripts for numeric data cleaning, cohort-specific feature selection based on prespecified missingness thresholds, exclusion of identifier and follow-up-time variables, zero imputation for selected binary or routinely documented variables, and development-cohort mean-based standardization and imputation for continuous variables.
- Model development and internal validation: Code for model development and comparison across the primary XGBoost-based model and baseline models, including XGBoost, random forest, logistic regression, ridge, lasso, and elastic-net logistic regression models. Internal performance was evaluated using stratified 5-fold cross-validation.
- Temporal external validation and performance evaluation: Scripts for evaluating model performance on the temporally distinct external validation cohort, including AUROC, sensitivity, specificity, positive predictive value, negative predictive value, F1 score, Brier score, bootstrap 95% confidence intervals, and paired bootstrap AUROC comparisons.
- Calibration analysis: Code for calibration intercept, calibration slope, observed-to-expected ratio, Brier score, and calibration assessment across risk strata.
- Decision-curve analysis: Code for estimating clinical net benefit across threshold probabilities, including treat-all and treat-none reference strategies.
- Model interpretability: SHAP-based analyses were implemented to estimate feature importance and generate SHAP summary and feature-importance visualizations, including bootstrap confidence intervals for mean absolute SHAP values.
- Subgroup analysis: Scripts for stratified performance evaluation across predefined clinical and demographic subgroups, including composite strata based on age, sex, and ADL deterioration.

Facility-level missingness analyses related to institution scales and regions were implemented in a separate modular script, as described in Multimedia Appendix 17.
