# Supplementary material for: Development and Temporal External Validation of a Parsimonious, Interpretable Machine Learning Model for Predicting 6-Month Mortality in Long-Term Care Facilities: A Retrospective Cohort Study
Source: JMIR Aging. 2026 Sep 16;9:e94567. doi: 10.2196/94567 (PMC13581260; doi:10.2196/94567)
Supplement: Supplementary file 18 [file aging-v9-e94567-s011.docx]

Figure S1. Decision Curve Analysis
